# Tracking Chromium Evolution on Ceria from Particles to Single Atoms: A Catalyst Regeneration Strategy for Ammonia Oxidation

**DOI:** 10.1021/jacs.5c08918

**Published:** 2025-11-30

**Authors:** Ivan Surin, Mikhail Agrachev, Frank Krumeich, Dragos Stoian, Qingxin Yang, Tatiana Otroshchenko, Jana Weiss, Christoph Kubis, Gunnar Jeschke, Evgenii V. Kondratenko, Javier Pérez-Ramírez

**Affiliations:** † Institute of Chemical and Bioengineering, Department of Chemistry and Applied Biosciences, 27219 ETH Zurich, Vladimir-Prelog-Weg 1, 8093 Zurich, Switzerland; ‡ Institute of Molecular Physics Science, Department of Chemistry and Applied Biosciences, ETH Zurich, Vladimir-Prelog-Weg 1, 8093 Zurich, Switzerland; § Laboratory of Inorganic Chemistry, Department of Chemistry and Applied Biosciences, ETH Zurich, Vladimir-Prelog-Weg 1, 8093 Zurich, Switzerland; ∥ Swiss Norwegian Beamlines, 55553 European Synchrotron Radiation Facility, Avenue des Martyrs 71, 38043 Grenoble, France; ⊥ Advanced Methods for Applied Catalysis, 28392 Leibniz-Institut für Katalyse, Albert Einstein-Str. 29a, 18059 Rostock, Germany

## Abstract

Supported chromium catalysts find broad application due to metal’s ability to assume a range of oxidation states and structures. However, the behavior of chromium species on reducible metal oxides under reactive conditions remains poorly understood, despite the potential for unique reactivity through metal–support interactions. Recent studies on CeO_2_-supported Cr^*n*+^ single atoms for selective NH_3_ oxidation to N_2_O underscore this potential, attaining high performance through cocatalytic action with CeO_2_, but also suffering from deactivation through agglomeration of isolated sites into Cr_2_O_3_. To address this challenge, we investigate how distinct CeO_2_-supported chromium species evolve under varying reactive environments. We show that under oxidative conditions redispersion of Cr_2_O_3_ occurs, serving as a catalyst regeneration strategy and enabling the recovery of both structure and performance. Combining advanced microscopy, in situ Raman, UV–vis, electron paramagnetic resonance and X-ray absorption spectroscopies, we follow the transformation from crystalline Cr_2_O_3_ nanoparticles to isolated chromium species. The redispersion is proposed to proceed via particle amorphization and oxidation of Cr^3+^ to mobile Cr^6+^, which diffuse over the support and stabilize as chromate species or as Cr^5+^ upon reduction by oxygen vacancies. A slower redispersion rate is observed over less reducible ZrO_2_ and TiO_2_, with minimal changes on γ-Al_2_O_3_ and Nb_2_O_5_, highlighting support reducibility as the driver of the process and reflecting the potential for generating Cr^6+^ sites from nontoxic Cr_2_O_3_ by controlling support properties. These results demonstrate how redox-active supports facilitate reversible changes to metal nanostructure, offering a promising strategy for regenerating catalysts and tuning metal speciation through rational support design for various applications.

## Graphical abstract

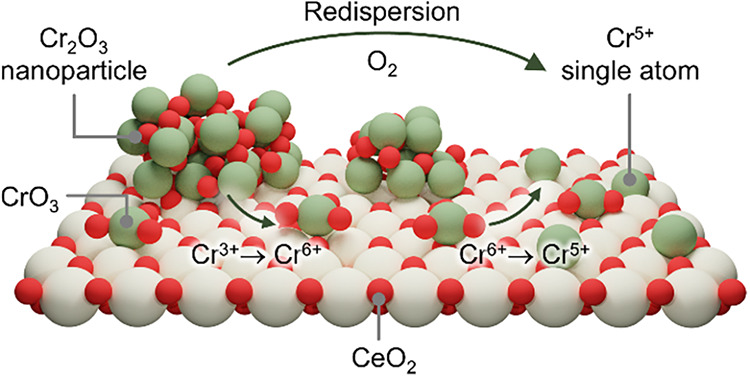

## Introduction

Supported chromium catalysts are essential for industrial chemistry, widely used for ethylene polymerization, alkane dehydrogenation and volatile organic compounds decomposition, with other applications continuing to emerge.^1–10^ The versatility of chromium in catalysis stems from its ability to assume a variety of oxidation states, while the metal speciation can range from crystalline metal oxide nanoparticles to clusters and single atoms. These factors directly govern catalyst activity and selectivity. Furthermore, as is common for supported catalysts, metal speciation can evolve under different reactive environments, which often leads to deactivation.^11^ Accordingly, there is strong interest in closely monitoring the structural dynamics in order to identify underlying drivers and develop methods for achieving, preserving or regenerating a desired nanostructure.^12–16^ In the case of chromium, numerous strategies, from support engineering to intricate catalyst treatment protocols, have been developed. However, most of these efforts have been focused on materials supported on nonreducible oxides, namely SiO_2_ and Al_2_O_3_,^1,3,4,17–24^ whereas reducible oxides, such as CeO_2_, ZrO_2_ and TiO_2_,^8,25–29^ have garnered significantly less attention. These support materials present an attractive avenue for further exploration, as their redox-active nature can enable simultaneous structural transformations of both the support and the metal phase, potentially giving rise to unique reactivity patterns.

Recently, chromium single atoms supported on CeO_2_ have emerged as a promising catalyst for selective ammonia, NH_3_, oxidation to nitrous oxide, N_2_O—offering a direct route to this selective oxidant.^30^ The oxygen vacancy-rich support provided a suitable surface to stabilize isolated Cr^*n*+^ sites, which synergized with CeO_2_ acting as a mediator of oxygen supply for N_2_O formation. However, under reaction conditions, single atoms agglomerated into Cr_2_O_3_ particles, which interact differently with the support and have been shown to favor undesired N_2_ formation.^31^ This behavior highlights how the dynamic structural evolution of the catalyst—affecting both the metal speciation and interaction with the support—can lead to catalyst deactivation. However, this also suggests that by systematically studying how distinct chromium structures on CeO_2_ evolve under different reactive conditions we could potentially identify strategies for controlling and restoring catalyst structure and performance. Beyond NH_3_ oxidation, such insights may aid in understanding the behavior of other catalytic systems involving metals on reducible supports and harsh redox reaction conditions, enabling the development of practical catalyst regeneration strategies.

Herein we study the behavior of chromium single atoms and Cr_2_O_3_ particles on CeO_2_ under different reactive environments. Remarkably, exposure of Cr_2_O_3_ to an oxidizing agent, i.e., O_2_, is found to trigger redispersion of Cr_2_O_3_ nanoparticles into isolated sites—a facile and effective method for regenerating the catalyst structure and regaining the initial NH_3_ conversion and N_2_O selectivity. Through a combination of advanced electron microscopy imaging, in situ Raman, UV–vis diffuse reflectance, electron paramagnetic resonance and X-ray absorption spectroscopies, we track the full redispersion process from crystalline Cr_2_O_3_ nanoparticles to single atoms. The process involves concurrent particle amorphization and oxidation of Cr^3+^ in Cr_2_O_3_ to mobile chromate, CrO_3_-like species, which migrate over the support surface and stabilize as either chromate species, or, to a significant extent, as Cr^5+^ upon reduction by oxygen vacancies. Similar structural transformations are observed over ZrO_2_ and TiO_2_, albeit at a lower rate than on CeO_2_, and to a limited extent over γ-Al_2_O_3_ and Nb_2_O_5_. This observation indicates that redispersion is governed by support reducibility, and, through careful control of synthetic parameters, enables the generation of Cr^6+^ sites on metal oxides from benign Cr_2_O_3_. These findings highlight the potential of redox-active supports to enable reversible structural transformations of the metal phase, offering a promising strategy for the design of regenerable catalysts with tunable speciation.

## Results and Discussion

### Materials Platform

In order to study the effects of different reactive environments on distinct chromium species, CeO_2_-supported chromium single atoms (Cr_SA_/CeO_2_) and Cr_2_O_3_ nanoparticles (Cr_2_O_3_/CeO_2_) were prepared following synthetic protocols schematically depicted in Figure 1a. X-ray diffraction (XRD) analysis of unsupported Cr_2_O_3_ nanoparticles was conducted, confirming the successful formation of crystalline Cr_2_O_3_ (Figure S2). However, no characteristic reflections of Cr_2_O_3_ were observed in the diffractogram of Cr_2_O_3_/CeO_2_, likely due to the low chromium content and the intense reflections of cubic CeO_2_. Nevertheless, the presence of different chromium species in the two samples was verified using high-angle annular dark field—scanning transmission electron microscopy (HAADF-STEM) coupled to energy-dispersive X-ray spectroscopy (EDXS, Figure 1b). In Cr_SA_/CeO_2_, chromium was found to be evenly dispersed across the CeO_2_ surface, whereas Cr_2_O_3_/CeO_2_ exhibited localized regions of high chromium concentration, characteristic of Cr_2_O_3_ nanoparticles. Additionally, continuous-wave electron paramagnetic resonance (CW-EPR) spectroscopy was performed, revealing distinct spectral features for each material (Figure 1c). The dominant feature in Cr_2_O_3_/CeO_2_ is a broad signal with *g* = 1.985, which is characteristic of antiferromagnetically superexchange-coupled Cr^3+^ ions in Cr_2_O_3_, consistent with the spectrum of Cr_2_O_3_ particles alone (Figure S3) and with previous studies.^25,32^ By contrast, an intense narrow signal with an axially anisotropic *g* tensor has been observed (*g*_⊥_ = 1.9635, *g*_∥_ = 1.9390) in the spectrum of Cr_SA_/CeO_2_. The *g* tensor is consistent with literature values for a similar Cr-doped CeO_2_ material^33^ and comparable to that of Cr dispersed on other supports,^25,34^ and can be attributed to paramagnetically isolated Cr^5+^ species. This is the only detectable feature for this sample, further evidencing the single-atom nature of chromium species. It should be noted that while the same narrow signal is also visible in the spectrum of Cr_2_O_3_/CeO_2_, it corresponds to only 0.2% of the total EPR signal, as determined by integration of the simulated spectrum (Figure S4). An in situ Raman spectroscopic analysis of Cr_SA_/CeO_2_ was also performed under dehydrated conditions to probe possible isolated Cr^6+^ sites, which are EPR-silent (Figure S5). The main identifiable chromium-related features of the spectra are the bands centered at ∼845 and ∼1015 cm^–1^. The former is characteristic of O–Cr–O stretching frequency in polychromates, specifically tetramers, while the latter is characteristic of stretching frequency in terminal Cr=O moieties of polychromates.^27,35^ Since the polychromate species are small and two-dimensional in nature, chromium dispersion is still likely to be full, and taking into account the fact that Cr^6+^ species likely constitute only a minor fraction of chromium in the sample,^30^ we can still expect single-atom chromium sites to be the dominant speciation in the sample.

**Figure 1:**
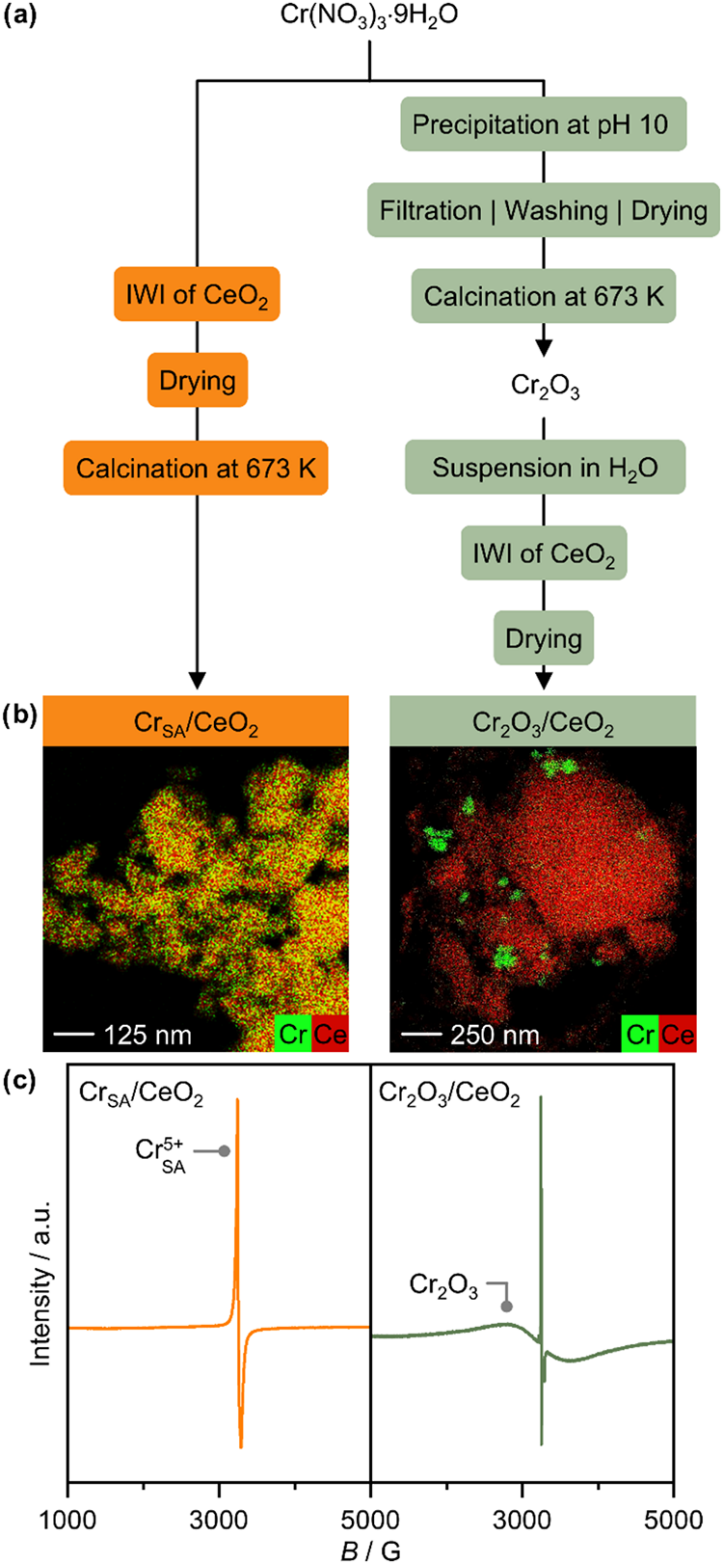
(a) Synthetic steps involved in the preparation of ceria-supported chromium catalysts with distinct metal speciation i.e., single-atom-based Cr_SA_/CeO_2_ and Cr_2_O_3_ nanoparticle-based Cr_2_O_3_/CeO_2_, the corresponding (b) EDX mappings, and (c) ex-situ EPR spectra acquired at room temperature.

### Effect of Reactive Environment on Chromium Structure

Before examining how individual reactants influence the structure of each catalyst, their performance in NH_3_ oxidation over an extended period was assessed (Figure S6). In line with the catalysts having different structures of chromium, they exhibited significant differences in initial performance and long-term trends (Figure S7). As previously reported,^30,31^ Cr_SA_/CeO_2_ suffered from deactivation over time on stream, primarily reflected in the decreasing rate of NH_3_ conversion, although a minor loss of N_2_O selectivity in favor of N_2_ could also be observed. Space-time yield of N_2_O, *STY*_N2O_, normalized per number of moles of chromium in the sample, similarly continuously decreases with time on stream, reflecting the fact that chromium is utilized less efficiently. Conversely, Cr_2_O_3_/CeO_2_ showed only a modest drop in NH_3_ conversion, while its N_2_O selectivity increased at the expense of N_2_. Still, the effect of rising N_2_O selectivity dominates and STY_N2O_ increases over time accordingly, until reaching a plateau. These observations suggest that both chromium nanostructures are sensitive to the conditions of NH_3_ oxidation and undergo a transformation during the reaction. In the case of Cr_SA_/CeO_2_, the deterioration of performance is likely due to agglomeration of single atoms into clusters or nanoparticles, as reported previously.^30^ However, the behavior observed for Cr_2_O_3_/CeO_2_ suggests that a different mechanism may be involved.

To gain a deeper understanding of the underlying reasons for these effects, the interaction of Cr_2_O_3_/CeO_2_ with individual reactants was investigated. Accordingly, in a series of experiments, the sample was subjected to treatment in flowing 8 vol % NH_3_ or 20 vol % O_2_ at the typical reaction temperature (i.e., 673 K), with periodic switching to the standard NH_3_ oxidation feed (i.e., 8 vol % NH_3_ + 8 vol % O_2_) to evaluate catalytic performance. This procedure is schematically depicted in Figure 2a. Interestingly, the performance of Cr_2_O_3_/CeO_2_ did not change following the treatment in NH_3_ flow (Figure 2b), reflected in virtually constant NH_3_ conversion, N_2_O selectivity and *STY*_N2O_. The likely reason for this is that Cr_2_O_3_ is not reducible under these conditions,^31^ and with the sample consisting primarily of large Cr_2_O_3_ nanoparticles, this reducing treatment is insufficient to modify chromium structure. In contrast, the treatment in O_2_ did result in a pronounced shift in catalytic behavior (Figure 2c): NH_3_ conversion experienced an initial dip, followed by a gradual increase and stabilization at a level slightly above the initial value. Even more strikingly, the catalyst displayed a consistent increase in N_2_O selectivity, accompanied by a corresponding decrease in N_2_ formation as the oxidative treatment progressed, until a stable level was achieved. Accordingly, despite the small initial dip in NH_3_ conversion, *STY*_N2O_ steadily increased. This trend was further confirmed under conditions of complete NH_3_ conversion, with selectivity changes being even more pronounced (Figure S8). Notably, the final selectivity, conversion and *STY*_N2O_ values reached by Cr_2_O_3_/CeO_2_ after the oxidative treatment closely match those of as-prepared Cr_SA_/CeO_2_, suggesting the presence of a similar chromium speciation in both materials. This implies that dispersion of Cr_2_O_3_ particles into isolated chromium sites could have occurred through interaction with O_2_. Importantly, these structural changes are not attributable to metal leaching or alterations in the porous structure of CeO_2_, as evidenced by elemental analysis with X-ray fluorescence and N_2_ sorption measurements (Table S1).

**Figure 2:**
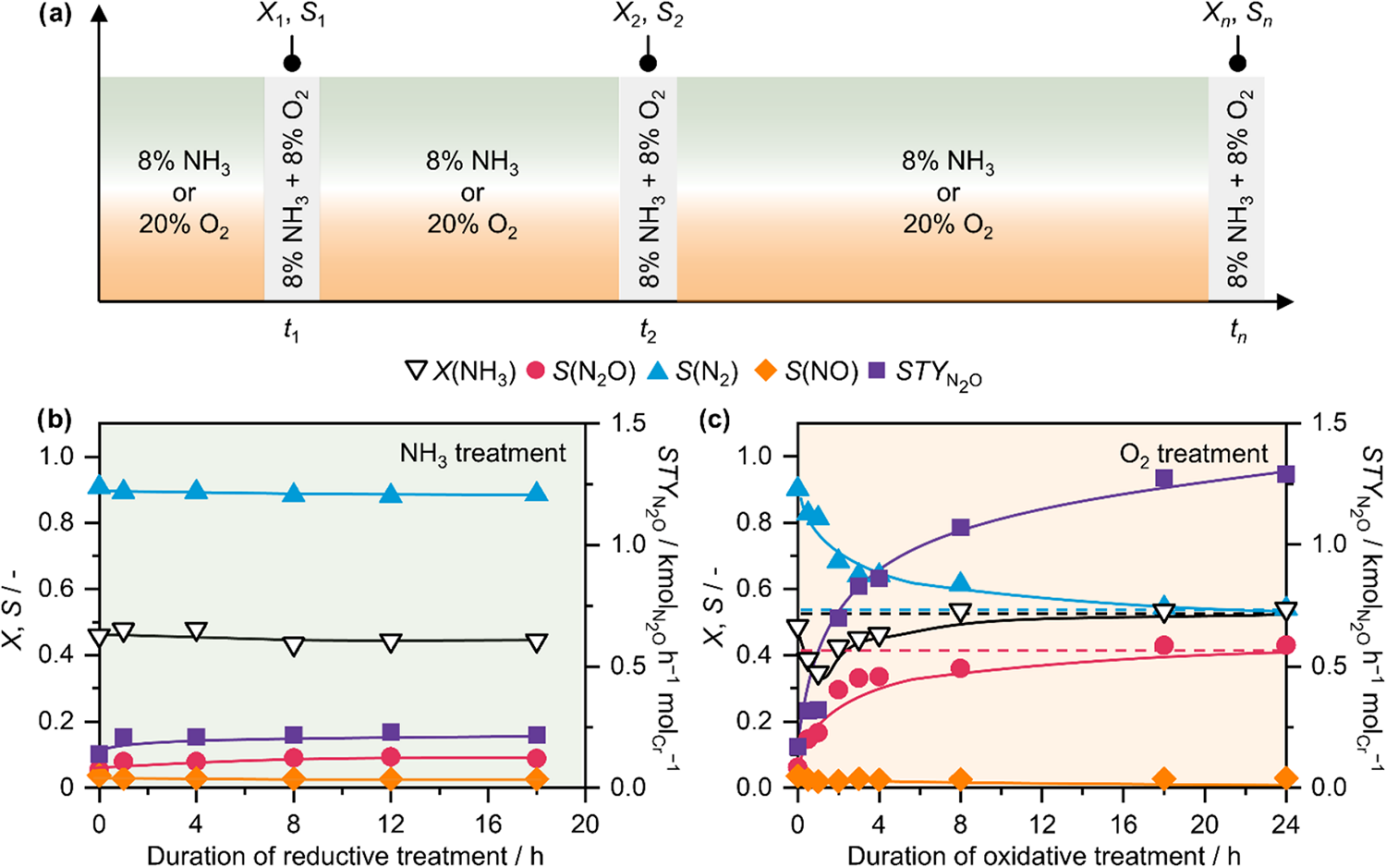
(a) Schematic representation of changes in reactive environment during a treatment of Cr_2_O_3_/CeO_2_ with NH_3_ or O_2_, with intermittent evaluation of catalytic activity; catalytic performance of Cr_2_O_3_/CeO_2_ in NH_3_ oxidation after variable duration of a (b) reductive treatment with NH_3_ or (c) oxidative treatment with O_2_. Dashed lines in (c) correspond to the initial performance of Cr_SA_/CeO_2_. Conditions: *T*_bed_ = 673 K; *m*_cat_ = 0.01 g; GHSV = 600,000 cm^3^ h^–1^ g_cat_^–1^; *P* = 1 bar; Feed (oxidative treatment): 20 vol % O_2_, 4 vol % Ar, 76 vol % He; Feed (reductive treatment): 8 vol % NH_3_, 4 vol % Ar, 88 vol % He.

Given that deactivation of Cr_SA_/CeO_2_ over time on stream in NH_3_ oxidation has been attributed to the agglomeration of chromium sites into Cr_2_O_3_ nanoparticles, redispersion of the latter via oxidative treatment presents a potential catalyst regeneration strategy. To verify this, a stability test was conducted, in which Cr_SA_/CeO_2_ was exposed to 16 h on stream, followed by 12 h of oxidative treatment. This cycle was repeated 3 times (Figure 3a). In line with the expectations, the initial level of NH_3_ conversion and *STY*_N2O_ could be almost fully restored, suggesting that efficiency of chromium utilization could be improved, while the rate of deactivation was not accelerated through repeated regeneration cycles. Elemental mapping further confirmed these findings: Cr_2_O_3_ nanoparticles were clearly visible on the catalyst surface after 16 h on stream but were no longer detectable immediately following the oxidative treatment (Figure 3b). These observations provide additional support for the conclusion that deactivation of Cr_SA_/CeO_2_ results from chromium agglomeration, which can be effectively reversed through controlled oxidative redispersion.

**Figure 3:**
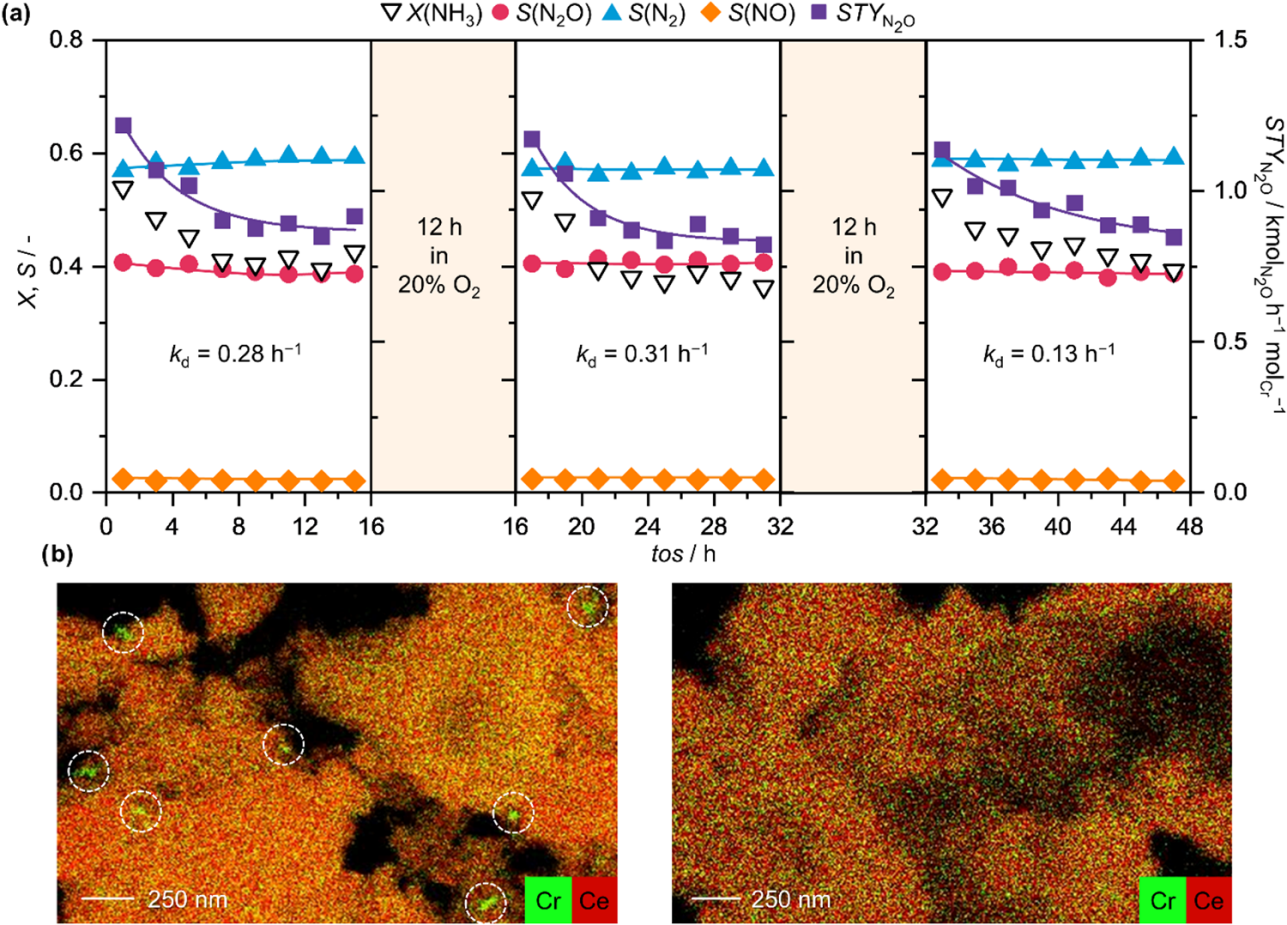
(a) Catalytic performance of Cr_SA_/CeO_2_ in NH_3_ oxidation, expressed in terms of NH_3_ conversion, product selectivity and space-time yield of N_2_O during a stability test with intermittent oxidative regeneration treatment. Deactivation constants, *k*_d_, have been obtained by fitting the *STY*_N2O_ data to a function of the type X = *C* – *e* ^–*k*_d_ *t*^; (b) EDX mappings of Cr_SA_/CeO_2_ after 16 h on stream (left) and directly after the regeneration treatment (right), showing the redispersion of Cr_2_O_3_ particles (circled) formed during the reaction. Conditions: *T*_bed_ = 673 K; *m*_cat_ = 0.01 g; GHSV = 600,000 cm^3^ h^–1^ g_cat_^–1^; *P* = 1 bar; Feed (reaction): 8 vol % NH_3_, 8 vol % O_2_, 4 vol % Ar, 80 vol % He; Feed (oxidative treatment): 20 vol % O_2_, 4 vol % Ar, 76 vol % He.

### Tracking Redispersion of Cr_2_O_3_

It should be noted that the Cr_2_O_3_ particles, shown in Figure 3b, still coexist with a large number of highly dispersed chromium species, and their formation in the presence of both reducing and oxidizing reactants may result in a structure that is easier to redisperse. Conversely, the particles in Cr_2_O_3_/CeO_2_ represent the only chromium nanostructure present in the catalyst and they were confirmed to be crystalline. Therefore, it should be verified whether the changes in catalytic performance observed in Figure 2c reflect a complete transformation from a nanoparticle-based to single-atom speciation of chromium. Accordingly, elemental mappings of Cr_2_O_3_/CeO_2_ after variable duration of the oxidative treatment were acquired (Figures 4 and S9). A gradual reduction in both the density and size of nanoparticles was observed, eventually leading to the absence of any clearly detectable aggregates after 24 h. However, it is important to note that while the number of measurable particles decreased, the estimated average particle size after 8 h of oxidative treatment remained nearly unchanged. This discrepancy is likely due to limitations inherent to EDXS and elemental mapping techniques, particularly for the system under study. Namely, Ce *L*_β1_ and Cr *K*_α_ emission lines are close in energy and, as a result, any Ce-containing region may appear to have well-dispersed chromium (Figure S10). Hence, the primary visual indicator for assessing chromium dispersion becomes the presence, or absence, of regions with highly concentrated Cr signal, typically associated with aggregates or Cr_2_O_3_ particles. However, beyond a certain particle size threshold or image resolution, reliably distinguishing these features becomes increasingly difficult. Therefore, in addition to microscopy imaging methods, complementary spectroscopic techniques are essential for tracking the redispersion process and gaining deeper insight into the evolving chromium structure.

**Figure 4:**
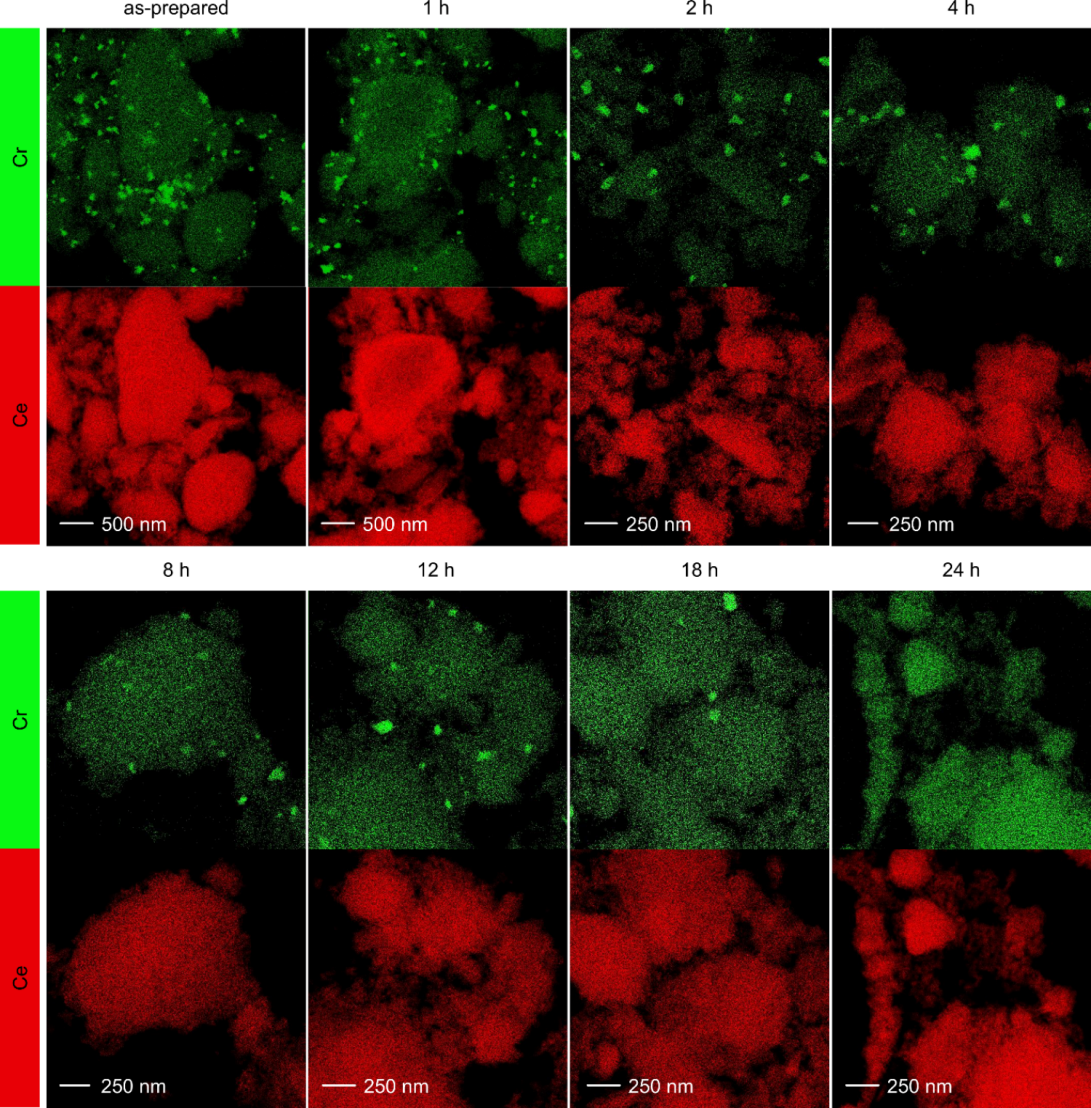
EDX mappings of Cr_2_O_3_/CeO_2_ after variable duration of an oxidative treatment.

### Spectroscopic Studies

To further probe the redispersion process, in situ Raman spectroscopy was employed due to its sensitivity to vibrational modes of different oxides of chromium and its well-established uses in the characterization of supported chromium species.^26,34,36^ It should be noted that the contacting pattern of O_2_ with the sample was found to influence the rate at which the redispersion of Cr_2_O_3_ takes place, with the transformation proceeding most rapidly in static air. This could be the consequence of the characteristic time of one of the initial steps of redispersion (e.g., O_2_ adsorption/dissociation) being longer than the contact time during a typical experiment in flow. Thus, while Cr_2_O_3_/CeO_2_ was dehydrated in flowing He prior to the in situ experiment, the oxidative treatment was performed in static air (Figure 5a). Additionally, due to the low overall metal loading, particularly of chromium species with distinct Raman signatures such as Cr^6+^, and the strong F_2g_ band at ∼466 cm^–1^ from the symmetric stretching vibration of Ce–O_8_ units in CeO_2_,^37^ the Raman bands associated with chromium appear relatively weak. A broad feature between 800 and 900 cm^–1^, centered at 845 cm^–1^, emerges shortly after the onset of oxidative treatment and grows steadily over time. This band has generally been assigned to O–Cr–O stretching modes in polychromate species, specifically tetramers.^27,36^ However, the absence of bands near 217 or 990 cm^–1^, characteristic of dimers and trimers, respectively, makes it difficult to confirm whether these species are also present. In addition, a band above 1000 cm^–1^, comprising two peaks at ∼1019 and ∼1034 cm^–1^, appears early in the treatment and increases in intensity as it proceeds. Both peaks are attributable to stretching vibrations of terminal Cr=O groups, with the ∼1019 cm^–1^ feature typical of polymeric species and the ∼1034 cm^–1^ feature characteristic of monomeric chromate units.^27,35^ Notably, no band at 555 cm^–1^, characteristic of Cr_2_O_3_ particles,^34^ was detected. This absence is likely due to the uneven distribution and relatively low concentration of Cr_2_O_3_ particles on the CeO_2_ surface, which increases the likelihood that the laser spot irradiated few or no particles, producing insufficiently strong signal. An additional contributing factor could be the presence of the CeO_2_ D band, associated with oxygen vacancies,^37^ in the 550–600 cm^–1^ region, potentially masking the Cr_2_O_3_ fingerprint. Nevertheless, microscopy imaging confirms the initial presence of Cr_2_O_3_ particles. Moreover, the progressive appearance of chromate-related bands during treatment provides further evidence for the mobility and dispersion of chromium species across the support surface.

**Figure 5:**
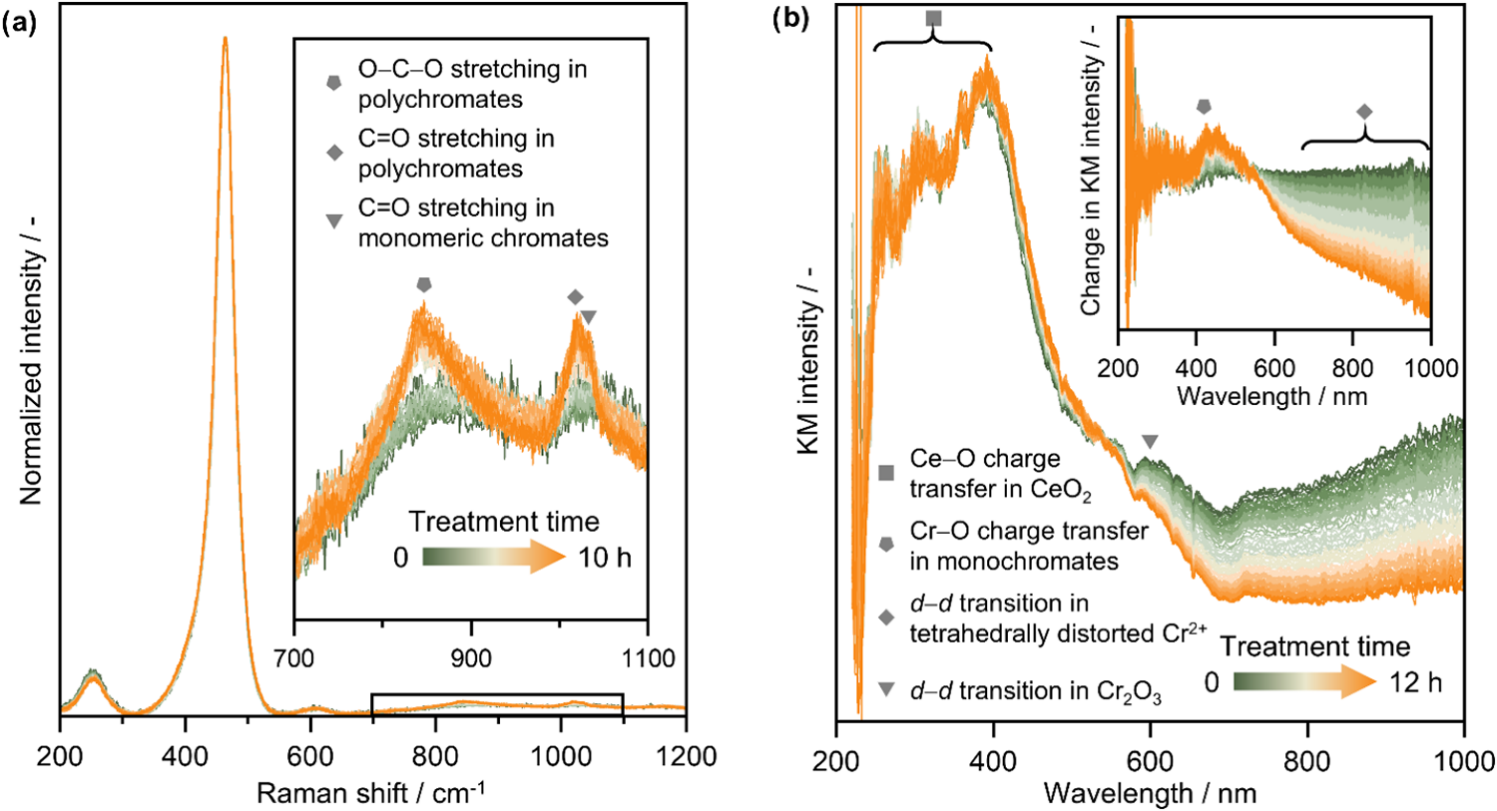
(a) In-situ Raman spectra of Cr_2_O_3_/CeO_2_ during a 10 h oxidative treatment in static air at 673 K. The region between 700 and 1100 cm^–1^ is magnified in the inset for better visibility of spectral changes. (b) In-situ UV–vis DRS spectra and difference spectra relative to the initial state of Cr_2_O_3_/CeO_2_ (inset) during a 12 h oxidative treatment in 20 vol % O_2_ in N_2_ at 673 K. Conditions: *T*_bed_ = 673 K; *m*_cat_ = 0.2 g; *F*_tot_ = 50 cm^3^ min^–1^; *P* = 1 bar; Feed: 20 vol % O_2_, 80 vol % N_2_.

Additionally, a slight decrease in the intensity of the band centered at ∼250 cm^–1^ was observed (Figure S11). This feature has been attributed to surface defect mode of O–Ce longitudinal stretching of atoms in the outermost layers of CeO_2_.^38^ It has also been shown to decrease in intensity upon surface reduction and to be an indicator for surface oxidation state of CeO_2_.^37^ Hence, the observed behavior during the redispersion treatment could serve as evidence that the oxidation of Cr^3+^ to Cr^6+^ occurs as a consequence of reaction between the Cr_2_O_3_ particles and CeO_2_, wherein oxygen vacancies are concurrently formed. This suggests that the interaction at the interface between CeO_2_ and Cr_2_O_3_ enables or at least facilitates the oxidation of Cr^3+^ in Cr_2_O_3_.

UV–visible diffuse reflectance spectroscopy (UV–vis DRS), another technique known to be sensitive to chromium speciation, was performed to gain further insight into the structural evolution of the catalyst. Ex-situ spectra of Cr_2_O_3_/CeO_2_, Cr_SA_/CeO_2_ and bare CeO_2_ revealed significant similarities in the 200–400 nm range, which can be primarily attributed to the strong absorption of CeO_2_ as a result of the O^2–^→Ce^3+/4+^ charge transfer transitions (Figure S12).^39^ Weak bands at 267 nm, in Cr_2_O_3_/CeO_2_, and at 357 nm, in both Cr_2_O_3_/CeO_2_ and Cr_SA_/CeO_2_, could potentially be attributed to O^2–^→Cr^6+^charge transfer in chromate species in tetrahedral coordination.^40^ However, their discernment from the CeO_2_ contributions is challenging. The spectrum of Cr_2_O_3_/CeO_2_ also exhibited two pronounced bands at 470 and 600 nm, associated with d–d transitions in Cr^3+^ in oxide clusters and in crystalline Cr_2_O_3_, respectively.^40^ In addition, a shoulder of a broad band above 700 nm could be observed, clearly present in Cr_2_O_3_/CeO_2_ but completely absent in other samples. However, it cannot be solely attributed to Cr_2_O_3_, as the reference spectrum of crystalline Cr_2_O_3_ shows no absorbance in this range.^18^ Literature reports have linked bands in the range of ∼800–1300 nm to d–d transitions in Cr^2+^ species in geometrically distorted environments.^34,41^ While existence of such species in bulk Cr_2_O_3_ is not expected, the strong metal–support interactions (SMSI) and redox properties of CeO_2_ could facilitate partial reduction of interfacial Cr atoms at nanoparticle periphery, producing the ill-defined shoulder we observe. In-situ UV–vis DRS analysis of Cr_2_O_3_/CeO_2_ during an oxidative treatment was also performed to see how the spectrum evolves over time (Figure 5b). A significant decrease in the intensity of all bands above 550 nm could be observed, with the spectrum gradually becoming more similar to that of Cr_SA_/CeO_2_. Features above 700 nm almost entirely disappeared, in agreement with the oxidation of chromium species. However, even after 12 h of treatment, a band near 600 nm decreased in intensity but did not fully disappear, indicating the continued presence of Cr_2_O_3_ particles—consistent with elemental mapping data (Figure 4). The difference spectrum (Figure 5b, inset) also reveals an increase in intensity at ∼455 nm. This band has been primarily attributed either to dichromate, or monochromate species.^24,42,43^ However, as Raman data (Figure 5a) do not support the presence of dimeric species and considering that metal–support interactions can shift band positions, we assign this feature to monomeric chromate moieties. In the context of ongoing redispersion, it is also plausible that the increase in this band could be obscuring the expected decrease in the band at 470 nm associated with Cr^3+^ in oxide clusters.^40^ This agrees with the results of Raman spectroscopy, suggesting that oxidation of Cr^3+^ to Cr^6+^ occurs during the oxidative treatment and further pointing to the involvement of chromate species in the redispersion process. Nevertheless, due to the low chromium content and the dominant absorption of CeO_2_ in the spectrally informative 200–400 nm region, further insights from UV–vis DRS are limited. Therefore, additional spectroscopic techniques are required to gain a more comprehensive understanding.

In-situ X-ray absorption spectroscopy was employed to track how the average oxidation state of chromium changes over time during oxidative treatment. The sample was placed in a quartz capillary with open ends, allowing for passive diffusion of air into and out of the tube. As this configuration did not permit heating the sample in an inert atmosphere, the sample was heated in air to 673 K at a rate of 1 K min^–1^ and held at this temperature for 8 h (Figure 6a). XAS spectra were collected approximately every 90 s throughout the treatment. However, due to the low chromium content and the resulting limited signal-to-noise ratio, the data could not be meaningfully interpreted on a frame-by-frame basis. Instead, the spectral evolution was visualized as a contour plot (Figure 6b) or analyzed by averaging over extended time intervals (Figure 6c).

**Figure 6:**
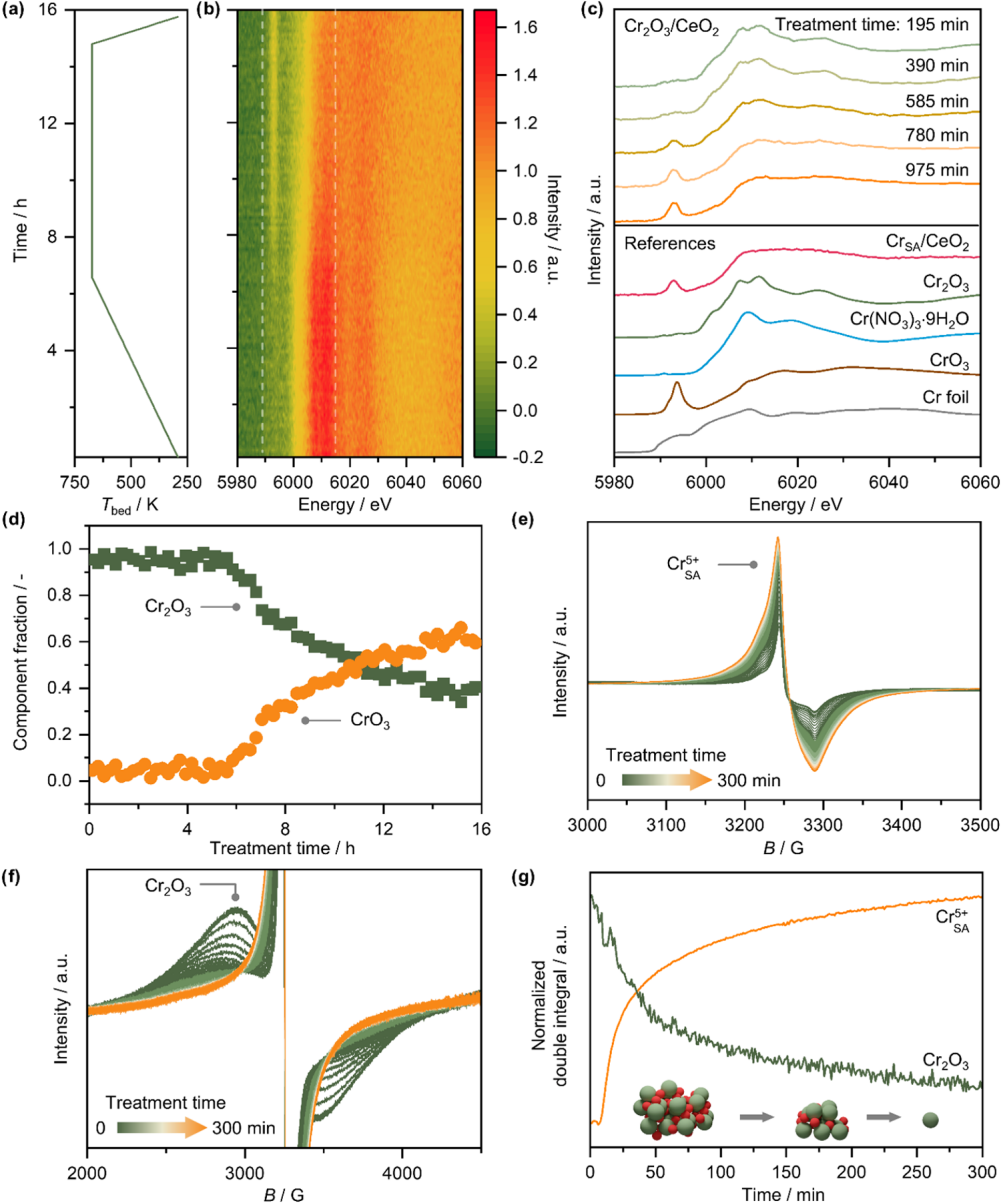
(a) Temperature profile of the catalyst bed during the in situ XAS experiment; (b) contour map of Cr *K* edge XANES spectra during the oxidative treatment of Cr_2_O_3_/CeO_2_; (c) averaged Cr *K* edge XANES spectra of Cr_2_O_3_/CeO_2_ at different points of the oxidative treatment and XANES spectra of reference materials; (d) concentration profile of the two main components present in Cr_2_O_3_/CeO_2_ during the oxidative redispersion treatment, identified using MCR-ALS, as a function of treatment time. (e, f) In-situ EPR spectra of Cr_2_O_3_/CeO_2_ during an oxidative treatment at 673 K. The increasing narrow signal in (e) and decreasing broad signal in (f) are indicative of the gradual disappearance of Cr_2_O_3_ particles, and concomitant increase in the concentration of magnetically isolated Cr^5+^ sites; (g) double integral of the Cr_2_O_3_ and Cr^5+^ EPR signals in (e) as a function of treatment time.

The contour map in Figure 6b shows virtually no changes until approximately 623 K, at which point the intensity of features between 6.005 and 6.015 keV begins to decline, marking the onset of a restructuring process. This observation aligns with the results from a temperature-programmed oxidation experiment of Cr_2_O_3_/CeO_2_ conducted in 20 vol % O_2_, wherein H_2_O formation could be detected starting around 600 K. The latter also suggests that dehydroxylation of CeO_2_ is likely to occur as a part of the redispersion process, which could be the mechanism by which chromium species get stabilized as isolated sites on the surface of CeO_2_ (Figure S13).

As the target temperature of 673 K is reached and the oxidative treatment continues, a further decrease in the intensity of features between 6.005 and 6.015 keV is observed. At the same time, a pre-edge feature at 5.993 keV emerges and grows in intensity. To interpret these spectral changes, the time-averaged spectra were analyzed and compared to reference standards with distinct chromium speciation (Figure 6c). The first two spectra represent averaged data acquired during the heating stage. Based on the lack of changes in Figure 6b in this time interval, they can be considered to closely represent the initial state of the catalyst, particularly the spectrum averaged over the first 195 min. As expected, this spectrum closely resembles the Cr_2_O_3_ standard, further confirming that the majority of chromium initially present in the sample exists as Cr_2_O_3_ nanoparticles. As the oxidative treatment progresses and chromium restructuring occurs, the shape of the averaged spectra becomes increasingly similar to the spectrum of Cr_SA_/CeO_2_, indicating that chromium speciation is transforming in favor of highly dispersed chromium sites. There are also prominent similarities to the CrO_3_ standard, with the pre-edge feature being especially notable, suggesting that Cr^6+^ species are forming. Still, while the available in situ XANES data provide strong evidence for the formation of Cr^6+^ species consistent with CrO_3_-like structures, the relatively low temporal resolution limits detailed tracking of how the speciation evolves. Complementary in situ measurements with higher time resolution, or supplementary techniques such as XPS could aid in further refining the speciation analysis of chromate species. In contrast, no features corresponding to Cr­(NO_3_)_3_·9H_2_O or metallic Cr foil were detected. Cr­(NO_3_)_3_·9H_2_O contains Cr^3+^ ions octahedrally coordinated by oxygen, serving as a potential proxy for isolated Cr^3+^ sites on the CeO_2_ surface, and its absence suggests that such species do not form in significant amounts during the treatment. Similarly, the lack of features associated with metallic Cr indicates that reduced chromium phases do not form under the oxidative conditions used.

To make the assessment of chromium speciation during the treatment more quantitative, the multivariate curve resolution—alternating least-squares (MCR-ALS) algorithm was applied to deconvolute the spectral data and extract individual contributing components, with Cr_2_O_3_ and CrO_3_ standards as the initial guesses. Upon convergence, two components closely resembling the spectra of these standards were identified, confirming that Cr_2_O_3_ and CrO_3_ are the predominant chromium species present during the treatment (Figure S14). When a third component was introduced, using the Cr­(NO_3_)_3_·9H_2_O spectrum as the initial guess, its contribution remained negligible when convergence was achieved (Figure S15). This indicates that isolated Cr^3+^ species, such as those represented by Cr­(NO_3_)_3_·9H_2_O, are not present in appreciable amounts during the oxidative treatment. The evolution of chromium speciation based on this analysis is presented in Figure 6d. It should be noted that while the rate of change in speciation slows over time, full stabilization is not achieved within the duration of the experiment, suggesting that structural transformation is still ongoing at the end point.

In-situ CW-EPR spectroscopy was also used to monitor the redispersion process. Similarly to the in situ XAS experiment, the oxidative treatment was performed in static air in an open capillary. However, due to differences in experimental setups, such as capillary dimensions, heating methods, and the amount of catalyst used, the rate of chromium restructuring differed, and thus the time scales of the two experiments are not directly comparable. Figures 6e,f show the evolution of in situ EPR spectra of Cr_2_O_3_/CeO_2_ over the course of 5 h. The broadening and decrease in intensity of the broad signal of CrO_*x*_ clusters (traditionally referred to as β-phase) from around 500 G to almost 1000 G can be seen in Figure 6f, which proceeds until its complete disappearance. The progressive broadening and decrease of this signal can be interpreted in the following way: for antiferromagnetic *α-*Cr_2_O_3_ particles line shape is determined by dipolar and exchange interactions between the Cr^3+^ ions.^44^ The former typically leads to line broadening and Gaussian lineshapes, while the latter, when the exchange constant *J* is large enough, leads to line narrowing and Lorentzian linseshape.^45^ For Cr_2_O_3_ particles and other Cr^3+^ clusters, Cr^3+^–Cr^3+^ exchange is typically strong, therefore Lorentzian lines appear with *g* ≅ 1.95 and peak-to-peak line widths of several hundred G, consistent with the signal observed here. The Néel temperature of bulk α-Cr_2_O_3_ is 307 K,^46^ therefore corresponding EPR signals are expected to be weak at room temperature and become stronger and narrower at higher temperatures, at which the material becomes paramagnetic, also in agreement with our observations. The Néel temperature, however, depends on the size, stoichiometry and presence of defects in the clusters, which consequently affect the characteristics and intensity of such signals. Moreover, the line width of such signals has been shown to decrease with increasing particles size.^47,48^ To show that this effect is also observable at our reaction temperature (673 K), we measured three model samples of Cr_2_O_3_ particles calcined at different temperatures (Figure S16). The spectra show a clear signal narrowing (from 850 to 450 G) and with increasing calcination temperature and hence particle size. This effect can be attributed to an increased exchange-narrowing due to an increase of exchange coupled spins. Moreover, the decrease in the fraction of surface uncompensated spins, which constitute the main contribution to the magnetization in antiferromagnetic nanoparticles, can also contribute to line narrowing. This phenomenon can be used to interpret the line shape changes observed in in situ measurements during oxidative redispersion: while the decrease of the double integral of CrO_*x*_ signal (Figure 6g) indicates a decrease in the number of particles, a concomitant line broadening takes place, which can be attributed to a decrease in particle size.

Concurrently, Figure 6e reveals a strong increase and broadening of the narrow signal associated with magnetically isolated Cr^5+^ sites. The latter effect is due to increasing dipole–dipole interactions as the density of isolated sites rises, resulting in shorter average distances between them. Furthermore, the increase in the double integral of the signal, which is directly proportional to the number of unpaired electrons in the isolated chromium ions, indicates that the number of such sites is increasing. Accordingly, the double integral of the Cr_2_O_3_ signal decreases at a similar rate over time (Figure 6g). These spectral changes are consistent with redispersion, wherein Cr^3+^ species in Cr_2_O_3_ transform into isolated Cr^5+^ sites. However, multiple spectroscopic techniques point to the involvement of chromate-like Cr^6+^ species in the redispersion process. It is therefore highly probable that the changes to chromium speciation observed in EPR spectra similarly proceed via initial oxidation of Cr^3+^ to Cr^6+^, followed by reduction to Cr^5+^ but we do not observe the intermediate stage because Cr^6+^ ions are diamagnetic and consequently EPR-silent.

While reduction of supported Cr^6+^ during calcination has been previously reported, it typically occurs at elevated temperatures.^26,36^ To verify whether CeO_2_ is capable of reducing Cr^6+^ under oxidative conditions, a new material, denoted as CrO_3_/CeO_2_, is synthesized through incipient wetness impregnation of CeO_2_ with an aqueous solution of CrO_3_ and subsequent calcination at 673 K. A comparison of the EPR spectra of Cr_SA_/CeO_2_ and CrO_3_/CeO_2_ revealed the presence of the sole signal of isolated Cr^5+^ sites, with similar peak area, suggesting that a substantial fraction of Cr^5+^ forms on CrO_3_/CeO_2_ through the reduction of Cr^6+^ (Figure S17). Additionally, the ex-situ XANES spectra of the two samples are highly comparable, further emphasizing that the electronic state of chromium is similar in the two samples (Figure S17).

### Influence of Support Reducibility on Chromium Redispersion

To investigate the influence of support reducibility on the redispersion Cr_2_O_3_ nanoparticles, monoclinic ZrO_2_, γ-Al_2_O_3_, TiO_2_ and Nb_2_O_5_ were used as supports, following the same synthetic protocol as for Cr_2_O_3_/CeO_2_, to yield Cr_2_O_3_/MO_*x*_ (MO_*x*_ = metal oxide). For comparison, the supports were impregnated with a solution of Cr­(NO_3_)_3_·9H_2_O to yield Cr/MO_*x*_ materials with high chromium dispersion, by analogy with Cr_SA_/CeO_2_. To assess whether redispersion is taking place, Cr_2_O_3_-based materials were calcined in static air at 673 K for 5 h and the catalytic performance of all materials in NH_3_ oxidation was evaluated (Figure S18). Notably, oxidative treatment of ZrO_2_- and TiO_2_-based samples has shifted their performance toward that of Cr/ZrO_2_ and Cr/TiO_2_, respectively. This is in line with the behavior observed for CeO_2_-supported samples and suggests that redispersion is likely to have taken place, to a significant extent over TiO_2_ and only partially over ZrO_2_. Conversely, no changes in performance of Al_2_O_3_- and Nb_2_O_5_-supported samples was observed, suggesting that the original nanoparticle speciation was preserved. To verify this, in situ EPR spectra during the oxidative treatment of each sample were collected (Figure S19). In the case of Cr_2_O_3_/ZrO_2_, there is a marked reduction in the broad Cr_2_O_3_ signal and a concurrent increase in the signal from magnetically isolated Cr^5+^ species, albeit with these changes occurring slower and to a lesser extent than in Cr_2_O_3_/CeO_2_. Cr_2_O_3_/TiO_2_ displayed similar spectral changes, slower than in Cr_2_O_3_/CeO_2_, but proceeding until the complete disappearance of the broad signal, unlike in Cr_2_O_3_/ZrO_2_. Conversely, Cr_2_O_3_/Al_2_O_3_ and Cr_2_O_3_/Nb_2_O_5_ showed little evidence of redispersion, with the spectra remaining largely unchanged during the treatment. To further emphasize the discrepancy between the two groups of catalysts, Cr_2_O_3_/ZrO_2_ and Cr_2_O_3_/Al_2_O_3_ were subjected to extended oxidative treatment, and the resultant materials were analyzed by microscopy. Elemental mappings of Cr_2_O_3_/ZrO_2_ show a progressive decrease in the number of particles as the duration of calcination is increased, accompanied by the increase in intensity of regions with highly dispersed chromium (Figure S20). After 20 h, the outline of the few remaining particles appear diffuse, signifying that the redispersion process is nearing completion. In contrast, Cr_2_O_3_/Al_2_O_3_ shows minimal signs of redispersion, even after 20 h of calcination (Figure S21). Only slight blurring of particle boundaries is observed, suggesting limited structural restructuring. These observations confirm that support reducibility strongly affects the extent and the rate of Cr_2_O_3_ redispersion under oxidative conditions, which is also supported by the temperature-programmed reduction with hydrogen (H_2_-TPR) profiles of individual metal oxide supports (Figure S22). Both TiO_2_ and ZrO_2_, while less reducible than CeO_2_, exhibit moderate surface redox activity. This activity likely facilitates initial oxidation of Cr^3+^ to Cr^6+^, as proposed for CeO_2_-supported system, and the appearance of the signal associated with isolated Cr^5+^ sites in in situ EPR spectra suggests that Cr^6+^ similarly get reduced, indicating that the same or highly similar redispersion mechanism is occurring on redox-active supports. On the other hand, Al_2_O_3_ is known to be nonreducible, while Nb_2_O_5_ requires much higher temperatures (>893 K) than the temperature of the oxidative treatment to activate surface redox sites, which results in neither of them being capable of facilitating the redispersion. Overall, oxidative treatment emerges as a general strategy for redispersing Cr_2_O_3_ particles over reducible oxides. By controlling the extent of the treatment, this approach could also be used to generate chromate species in situ, eliminating the need to handle toxic Cr^6+^ precursors.

## Conclusion

This study explored and followed the dynamic structural transformations of chromium supported on CeO_2_ and other metal oxides in varying reactive environments. Systems comprising single chromium atoms and Cr_2_O_3_ nanoparticles were investigated. NH_3_ oxidation to N_2_O was adopted as a structure-sensitive reaction, previously shown to be effectively catalyzed by CeO_2_-supported chromium single atoms, which, however suffered from deactivation. We demonstrate that the loss of activity and N_2_O selectivity, associated with the transformation of isolated sites into Cr_2_O_3_, could be reversed by means of an oxidizing treatment, enabling catalyst regeneration and recovery of performance. This restructuring process was tracked in detail using microscopic and spectroscopic techniques, capturing the evolution from crystalline Cr_2_O_3_ nanoparticles down to isolated Cr^*n*+^ species (Figure 7). The proposed mechanism involves oxidation of Cr^3+^ in Cr_2_O_3_ particles to Cr^6+^, which can diffuse across the metal oxide surface and either anchor directly as chromate species or be reduced by the redox-active support and stabilized as Cr^5+^ species. The reducibility of the support was found to play a critical role in determining the rate and extent of redispersion: the process proceeded significantly more slowly over the less reducible ZrO_2_ and TiO_2_ and was virtually absent on nonreducible Al_2_O_3_ and Nb_2_O_5_. This work highlights how redox-active supports enable reversible changes in metal speciation, offering a pathway to regenerable catalysts. Notably, the findings suggest that oxidative redispersion, already reported for noble metals such as Pt and Pd, may be more broadly applicable to metal oxide systems.^12^ By leveraging support properties to facilitate such transformations, this approach opens new possibilities for designing robust, tunable catalysts with regeneration potential for a wide range of redox reactions.

**Figure 7:**
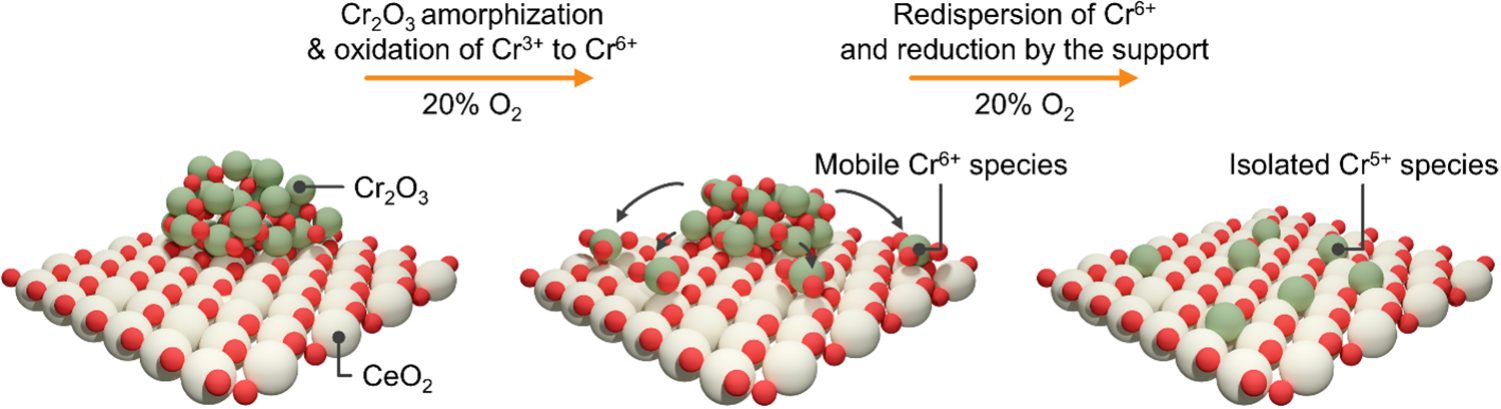
Schematic representation of the structural evolution of chromium species during an oxidative treatment, which triggers particle redispersion.

## Supplementary Material



## Data Availability

The experimental data presented in the main figures of the manuscript are publicly available through Zenodo (10.5281/zenodo.15518602).

## Notes

Experimental procedures, i.e., catalyst preparation methods, catalyst characterization methods, catalyst evaluation methods (PDF)
