# Supplementary material for: Tracking Chromium Evolution on Ceria from Particles to Single Atoms: A Catalyst Regeneration Strategy for Ammonia Oxidation
Source: J Am Chem Soc. 2025 Nov 30;147(49):44759–69. doi: 10.1021/jacs.5c08918 (PMC12703660; doi:10.1021/jacs.5c08918)
Supplement: Supplementary file 1 [file ja5c08918_si_001.pdf]

Supporting Information

Tracking Chromium Evolution on Ceria from Particles to Single Atoms: A Catalyst Regeneration Strategy for Ammonia Oxidation

Ivan Surin,[1] *Mikhail Agrachev,*[2] *Frank Krumeich,*[3] *Dragos Stoian,*[4] *Qingxin Yang,*[1] *Tatiana Otroshchenko,*[5] *Jana Weiss,*[5] *Christoph Kubis,*[5] *Gunnar Jeschke,*[2] *Evgenii V. Kondratenko*[5] *and Javier Pérez-Ramírez*[1] *

[1] Institute of Chemical and Bioengineering, Department of Chemistry and Applied Biosciences, ETH Zurich, Vladimir-Prelog-Weg 1, 8093 Zurich, Switzerland.

[2] Institute of Molecular Physics Science, Department of Chemistry and Applied Biosciences, ETH Zurich, Vladimir-Prelog-Weg 1, 8093 Zurich, Switzerland.

[3] Laboratory of Inorganic Chemistry, Department of Chemistry and Applied Biosciences, ETH Zurich, Vladimir-Prelog-Weg 1, 8093 Zurich, Switzerland.

[4] Swiss Norwegian Beamlines, European Synchrotron Radiation Facility, Avenue des Martyrs 71, 38043 Grenoble, France.

[5] Advanced Methods for Applied Catalysis, Leibniz-Institut für Katalyse, Albert Einstein-Str. 29a, 18059 Rostock, Germany.

* Corresponding author. E-mail: jpr@chem.ethz.ch

Table of contents

1. Experimental procedures 2

1.1. Catalyst synthesis 2

1.2. Catalyst characterization 2

1.3. Catalyst evaluation 4

2. Supplementary tables 6

3. Supplementary figures 7

4. Supplementary references 25

1. Experimental procedures

1.1. Catalyst synthesis

Chromium oxide, Cr_2O_3, was synthesized *via* a precipitation method. 5 g of chromium nitrate $Cr(NO_3)_3 \cdot 9H_2O$ (Sigma Aldrich, 99%) was dissolved in 80 cm^3 of water. Under vigorous stirring, 2 M aqueous NH_3 solution was added dropwise until a pH of 10 was reached. The suspension was stirred for 24 h, the filtrate was washed with at least 1 L of deionized water and dried overnight in a vacuum oven at 353 K. The dried powder was calcined in static air at 673, 873 or 1073 K (heating rate = 3 K min^{-1}, hold time = 5 h).

Cerium oxide, CeO_2, was synthesized *via* thermal decomposition of $Ce(NO_3)_3 \cdot 6H_2O$ (Sigma Aldrich, 99%) in static air at 673 K (heating rate = 3 K min^{-1}, hold time = 5 h). The resulting solid was ground into powder in a mortar.

Supported nanoparticle-based chromium catalysts on various carriers (Cr_2O_3/support, support = CeO_2 (specific surface area, S_{BET} = 83 m^2 g^{-1}), ZrO_2 (S_{BET} = 50 m^2 g^{-1}), Al_2O_3 (S_{BET} = 60 m^2 g^{-1}), TiO_2 (S_{BET} = 52 m^2 g^{-1}), Nb_2O_5 (S_{BET} = 28 m^2 g^{-1})) were synthesized *via* incipient wetness impregnation (IWI) with a nominal Cr loading of 1 wt%. Accordingly, the Cr_2O_3 particles calcined at 673 K were added to an appropriate amount of water, the resulting suspension was sonicated for 10 min and subsequently added dropwise to the respective support, including CeO_2, ZrO_2 (Sigma-Aldrich, nanopowder < 100 nm), Al_2O_3 (Alfa Aesar, 99.98%), TiO_2 (Sigma Aldrich, nanopowder, 99.5%) and Nb_2O_5 (Sigma Aldrich, 99.99%). After impregnation, all the samples were dried overnight under vacuum at 353 K.

Supported single-atom-based chromium catalysts on CeO_2 (Cr_{SA}/CeO_2, CrO_3/CeO_2) were synthesized *via* IWI method with a nominal Cr loading of 1 wt%. Accordingly, $Cr(NO_3)_3 \cdot 9H_2O$ (Sigma Aldrich, 99%) or CrO_3 (Thermo Scientific, 99%) was dissolved in deionized water and the resulting solution was added dropwise to the support. After impregnation, all samples were dried overnight under vacuum at 353 K and then calcined in static air at 673 K (heating rate = 3 K min^{-1}, hold time = 5 h). Highly dispersed chromium catalysts on ZrO_2, Al_2O_3, TiO_2 and Nb_2O_5 were synthesized following analogous procedure, using $Cr(NO_3)_3 \cdot 9H_2O$ as the precursor.

1.2. Catalyst characterization

Powder X-ray diffraction (XRD) measurements were conducted on Rigaku SmartLab diffractometer using Cu-Kα radiation (λ = 0.1541 nm). The data was recorded in the range of 10-70° 2θ with an angular step size of 0.017° and a counting time of 0.26 s per step.

N_2 adsorption-desorption measurements were performed at 77 K in a Micrometrics TriStar II instrument. The sample (catalyst mass, m_{cat} = 0.1-0.2 g; particle size, d_p = 0.2-0.4 mm) was degassed at 473 K for 3 h prior to the measurement.

Temperature-programmed reduction with H_2 and temperature-programmed oxidation was performed in a Micromeritics Autochem II coupled to a Pfeiffer Vacuum OmniStar mass spectrometer (H_2-TPR,

TPO-MS). Prior to the measurement, the sample (m_{cat} = 0.1 g; d_p = 0.2-0.4 mm) was loaded into a U-shaped quartz micro-reactor, dried in He at 673 K (total volumetric flow rate, F_T = 20 cm^3 min^{-1}, heating rate = 10 K min^{-1}) for 30 min, and then cooled down to 313 K (cooling rate = 20 K min^{-1}). The TPO was then performed under flowing 10 vol% O_2 in He (F_T = 20 cm^3 min^{-1}) by heating the sample to 673 K (heating rate = 10 K min^{-1}, hold time = 5 h), with the evolution of H_2O (m/z = 18) monitored by the MS. The TPR was then performed under flowing 5 vol% H_2 in Ar (F_T = 20 cm^3 min^{-1}) by heating the sample to 1073 K (heating rate = 10 K min^{-1}, hold time = 5 h), with H_2 consumption monitored by a thermal conductivity detector.

High-angle annular dark-field scanning transmission electron microscopy (HAADF-STEM) micrographs and energy-dispersive X-ray spectroscopy (EDXS) mappings were acquired on a JEOL JEM-F200 microscope with a cold field emission gun operated at an acceleration potential of 200 kV. The EDX system of this microscope is comprised of 2 silicon drift detectors (SDD) which enable the recording of EDXS maps with good signal-to-noise ratio in a relatively short collection time (5-15 min).

In-situ Raman spectroscopy was performed on a Renishaw inVia Raman microscope using a air-cooled He:Ne 633 nm laser, with a laser power of 8.5 mW and 20× objective. The catalyst (m_{cat} = 0.05 g; d_p = 0.2-0.4 mm) was loaded into a ceramic Linkam reaction cell (CCR1000), connected to a gas supplying system. Prior to the thermal treatments a mapping was conducted at room temperature collecting spectra at 15-16 different spots on the samples. Prior to in-situ oxidative treatment, the sample (Cr_2O_3/CeO_2, **Figure 5a**) was dried at 673 K in flowing He (F_T = 20 cm^3 min^{-1}, heating rate = 10 K min^{-1}, hold time = 30 min), after which the gas inlet was disconnected to allow ambient air to diffuse freely into the system. Spectra were subsequently collected every 15 min for 10 h with acquisition time of 10 s, and accumulation number of 1. For Cr_{SA}/CeO_2 (**Figure S5**), the sample was dried at 473 K in flowing He (F_T = 20 cm^3 min^{-1}, heating rate = 10 K min^{-1}, hold time = 30 min), while spectra were collected every 5 min with acquisition time of 10 s, and accumulation number of 1.

In situ diffuse reflectance UV-vis spectroscopy (UV-vis DRS) was performed using an AVASPEC fiber optical spectrometer (Avantes) equipped with a deuterium-halogen light source (AvaLight-DH-S-BAL) and a CCD array detector. The details of the UV-vis apparatus were described in elsewhere. $BaSO_4$ was used as a white reference material. The catalyst (m_{cat} = 0.2 g; d_p = 0.2-0.4 mm) was loaded into a quartz reactor (internal diameter = 6 mm). The catalyst bed was fixed between two layers of quartz wool. A high-temperature reflection probe including six light fibers and one reading fiber was positioned perpendicular to the reactor. Ex situ spectra were acquired. The catalyst was then heated to 673 K in flowing N_2 (F_T = 10 cm^3 min^{-1}, heating rate = 10 K min^{-1}). Upon switching the gas composition to 20 vol% O_2 in N_2 (F_T = 50 cm^3 min^{-1}), UV-vis spectra (from 200 to 800 nm) were recorded for 12 h, acquiring a spectrum every 1 min during the first hour and every 5 min thereafter. Using the time-resolved reflectance R values, Kubelka-Munk function $F(R)$ was calculated according to **Equation 1**.

$$F(R) = \frac{(1-R)^2}{2R} \quad (1)$$

Continuous-wave electron paramagnetic resonance (CW-EPR) spectroscopy experiments were conducted on a Bruker Elexsys E500 spectrometer operating at X band frequencies, using an ER4102ST microwave resonator and equipped with an Oxford helium (ESR900) cryostat. All CW-EPR spectra were acquired at room temperature with the following spectrometer parameters: microwave frequency = 9.8 GHz, sweep width = 590 mT, center field = 300 mT, modulation frequency = 100 kHz, modulation amplitude = 3 G, microwave power = 2.012 mW, power attenuation = 20 dB, conversion time = 327.68 ms. All measured g factors were offset corrected against a known standard (*i.e.*, free radical 1,1-diphenyl 2-picrylhydrazyl). In situ electron paramagnetic resonance (EPR) spectroscopy experiments were performed using a custom-built setup. The catalyst (m_{cat} = 50 mg; d_p = 0.2-0.4 mm) was loaded into a quartz capillary (internal diameter, d = 0.8 mm) and placed inside an EPR quartz tube (d = 2.8 mm, Wilmad). The EPR tube was housed at the center of a homemade water-cooled high-temperature resonator, which was installed into a continuous wave (CW) EPR spectrometer (Bruker EMX) operating at X-band frequencies. EPR spectra were recorded at room temperature in flowing He (F_T = 50 cm^3 min^{-1}). Subsequently, the reactor was heated to the desired temperature (T = 673 K) and EPR spectra were continuously recorded until no changes were observed. Next, for in-situ tests with varying feed composition, the corresponding gas mixture (8 vol% NH_3, 8 vol% O_2, and 84 vol% He; 8 vol% NH_3 and 92 vol% He; or 8 vol% O_2 and 92 vol% He) was fed at a total volumetric flow of F_T = 50 cm^3 min^{-1}, continuously recording EPR spectra until no changes were observed. Alternatively, for oxidative treatment, gas feed and outlet were disconnected, and the sample was kept in static air, continuously recording EPR spectra until no changes were observed.

X-ray absorption spectroscopy (XAS) measurements were performed at the Swiss-Norwegian beamlines (SNBL, BM31) at the European Synchrotron Radiation Facility (ESRF).[1] A continuous scan mode was employed using a Si(111) LN_2-cooled double crystal monochromator. The unfocussed beam size at the sample position was *ca.* 3 mm × 0.300 mm H. Cr metal foil (*K*-edge at 5989.2 eV, measured in transmission using ion chambers) was employed as a standard for energy calibration. The measurements were performed in fluorescence geometry using a Vortex SDD with XIA Falcon X fast digital multichannel analyzer electronics as detection system (*ca.* 90 s per XANES scan). Under air atmosphere, the sample (m_{cat} = 50 mg) was loaded into a quartz capillary (d = 0.7 mm, wall thickness = 0.01 mm, Hilgenberg GmbH); samples were carefully packed from both sides using quartz wool and heated at a rate of 1 K min^{-1} to 673 K.[2] After careful data examination, no corrections were applied to the results acquired in fluorescence mode, as the data were similar in terms features (intensity and position) with the measurements made in transmission mode on relevant standards. Data reduction protocols *i.e.*, background removal, normalization and averaging (scans were averaged for better signal-to-noise ratio), truncation/deglitching, etc. were performed using Athena software from the Demeter package.[3] Lastly, principal component analysis (PCA) together with multivariate curve

resolution with alternating least squares algorithm (MCR-ALS) were used as a dimensionality reduction method for simplification of larger dataset to a minimum number of relevant components and a powerful engine for the extraction of kinetically and spectroscopically pure component spectra and their corresponding concentration profiles. More details on the protocols and data analysis software have been reported elsewhere.[4]

1.3. Catalyst evaluation

Evaluation of catalytic performance in continuous-flow NH_3 oxidation was conducted in a fixed-bed micro-reactor (**Figure S1**). All experiments were conducted at atmospheric pressure. The flow rate of gases, He (PanGas, purity 4.6, diluent), NH_3 (PanGas, purity 3.8), O_2 (PanGas, purity 5.0) and Ar (PanGas, purity 5.0, internal standard) was regulated using thermal mass-flow controllers (Bronkhorst), connected to a mixing unit equipped with a pressure gauge. The catalyst (m_{cat} = 0.01–0.2 g; d_p = 0.2-0.4 mm; for tests at elevated gas-hourly space velocity, $GHSV$ > 15,000 $cm^3\ h^{-1}\ g_{cat}^{-1}$, the catalyst bed was diluted with silicon carbide (d_p = 0.5-0.6 mm) to avoid the formation of hot spots) was loaded into a quartz micro-reactor (d = 8 mm or 2 mm), containing a bed made of quartz wool and placed in an electrical oven. The temperature in the middle of the catalyst bed was monitored and controlled using a K-type thermocouple placed in a coaxial quartz thermowell. Prior to testing, the catalyst was heated in a He flow (T_{bed} = 473 K, F_T = 50 $cm^3\ min^{-1}$) for 30 min, subsequently heated to the desired temperature (T_{bed} = 673 K) and allowed to stabilize for at least 30 min. For evaluation of catalytic performance, the reaction mixture (8 vol% NH_3, 8 vol% O_2, 4 vol% Ar, and 80 vol% He) was fed at a total volumetric flow of F_T = 50-100 $cm^3\ min^{-1}$.

Nitrogen-containing compounds (NH_3, N_2, N_2O, NO_2 and NO), as well as O_2 and Ar were quantified *via* an online gas chromatograph equipped with a GS CP-Volamine column coupled to a mass spectrometer (GC-MS, Agilent, GC 7890B, MSD 5977A). Upon acquisition of the full chromatogram, individual ion chromatograms at *m*/*z* 17, 28, 30, 32, 40, 44 and 46 were extracted. A single peak was observed on chromatograms at *m*/*z* 32, 40 and 44, allowing to directly quantify O_2, Ar, and N_2O, respectively. Sufficiently different retention times of N_2O (t = 1.966 s), N_2 (t = 1.887 s) and NO (t = 1.892 s) allowed resolution of the peaks attributed to N_2O fragments, N_2 and NO in the product stream at *m*/*z* 28 and 30, so that subsequent quantification of N_2 and NO could be performed. Sufficiently different retention times of NH_3 (t = 1.985 s) and H_2O (t = 2.065 s) allowed resolution of their respective peaks at *m*/*z* 17 and quantification of NH_3.

The conversion of NH_3 was calculated according to **Equation 2**,

$$X_{NH_3} = \frac{\dot{n}_{NH_3}^{in} - \dot{n}_{NH_3}^{out}}{\dot{n}_{NH_3}^{in}} \qquad (2)$$

where $\dot{n}_{NH_3}^{in}$ and $\dot{n}_{NH_3}^{out}$ denote the molar flows of NH_3 at the reactor inlet and outlet, respectively. Selectivity towards a product i was determined according to **Equation 3**,

$$S_i = \frac{\nu_i \dot{n}_i^{\text{out}}}{\dot{n}_{\text{NH}_3}^{\text{in}} X_{\text{NH}_3}} \tag{3}$$

where ν_i is the number of N atoms in the product molecule (*i.e.*, $\nu = 2$ for N_2O or N_2, and $\nu = 1$ for NO). The space-time yield of N_2O (STY_{N2O}) was calculated according to **Equation 4**,

$$STY_{\text{N}_2\text{O}}(\text{mol}_{\text{N}_2\text{O}}\,\text{h}^{-1}\,\text{mol}_{\text{Cr}}^{-1}) = \frac{\dot{n}_{\text{NH}_3}^{\text{in}} \cdot X_{\text{NH}_3} \cdot S_{\text{N}_2\text{O}}}{n_{\text{Cr}}} \tag{4}$$

where n_{Cr} is the number of moles of chromium in the sample. Nitrogen (B_{N}) and oxygen (B_{O}) balances were evaluated for each catalytic test according to **Equation 5** and **Equation 6**, respectively:

$$B_{\text{N}}(\%) = \frac{\dot{n}_{\text{NH}_3}^{\text{out}} + 2\dot{n}_{\text{N}_2\text{O}}^{\text{out}} + 2\dot{n}_{\text{N}_2}^{\text{out}} + \dot{n}_{\text{NO}}^{\text{out}}}{\dot{n}_{\text{NH}_3}^{\text{in}}} \times 100 \tag{5}$$

$$B_{\text{O}}(\%) = \frac{\dot{n}_{\text{O}_2}^{\text{out}} + 2\dot{n}_{\text{N}_2\text{O}}^{\text{out}} + 1.5\dot{n}_{\text{N}_2}^{\text{out}} + 1.25\dot{n}_{\text{NO}}^{\text{out}}}{\dot{n}_{\text{O}_2}^{\text{in}}} \times 100 \tag{6}$$

The error of the B_{O} was less than 5% in all experiments. After the tests, the reactor was quenched to room temperature in He flow and the catalytic materials were retrieved for studies.

2. Supplementary tables

Table S1. Sample code, porous properties and metal content of the catalysts studied in this work.

Catalyst	S_{BET}[f] / $m^2\ g^{-1}$	Total pore volume[f] / $cm^3\ g^{-1}$	Metal content[g] / wt%
Cr_2O_3/CeO_2[a]	82	0.15	0.76
Cr_2O_3/CeO_2-1h[b]	80	0.15	0.76
Cr_2O_3/CeO_2-2h	83	0.14	0.80
Cr_2O_3/CeO_2-4h	74	0.14	0.63
Cr_2O_3/CeO_2-8h	81	0.14	0.74
Cr_2O_3/CeO_2-12h	75	0.14	0.76
Cr_2O_3/CeO_2-18h	80	0.14	0.73
Cr_2O_3/CeO_2-24h	73	0.14	0.83
Cr_{SA}/CeO_2[c]	80	0.15	0.95
CrO_3/CeO_2[d]	78	0.14	0.92
Cr/ZrO_2[c]	50	0.16	0.98
Cr_2O_3/ZrO_2[a]	49	0.16	0.91
Cr_2O_3/ZrO_2-C673-5h[e]	51	0.16	0.92
Cr_2O_3/ZrO_2-C673-10h[e]	52	0.16	0.81
Cr_2O_3/ZrO_2-C673-20h	51	0.16	0.91
Cr/Al_2O_3[c]	56	0.21	0.90
Cr_2O_3/Al_2O_3[a]	58	0.21	0.81
Cr_2O_3/Al_2O_3-C673-5h[e]	58	0.20	0.83
Cr_2O_3/Al_2O_3-C673-10h[e]	60	0.20	0.81
Cr_2O_3/Al_2O_3-C673-20h	55	0.20	0.81
Cr/TiO_2[c]	52	0.21	1.01
Cr_2O_3/TiO_2[a]	49	0.20	0.90
Cr_2O_3/TiO_2-C673-5h[e]	47	0.20	0.91
Cr/Nb_2O_5[c]	28	0.12	0.85
Cr_2O_3/Nb_2O_5[a]	27	0.12	0.91
Cr_2O_3/Nb_2O_5-C673-5h	28	0.11	0.90

[a] Prepared *via* incipient wetness impregnation with an aqueous suspension of Cr_2O_3, followed by drying in vacuum only; [b] '-*X*h' suffix refers to the duration of the oxidative treatment (*X* hours) in flowing 20 vol% O_2 at 673 K; [c] Prepared *via* incipient wetness

impregnation with an aqueous solution of $Cr(NO_3)_3 \cdot 9H_2O$, followed by calcination at 673 K; [d] Prepared *via* incipient wetness impregnation with an aqueous solution of CrO_3, followed by calcination at 673 K; [e] '-C673-*X*h' suffix refers to the duration of calcination (*X* hours) in static air at 673 K; [f] Determined by N_2 sorption; [g] Determined by XRF analysis.

3. Supplementary figures

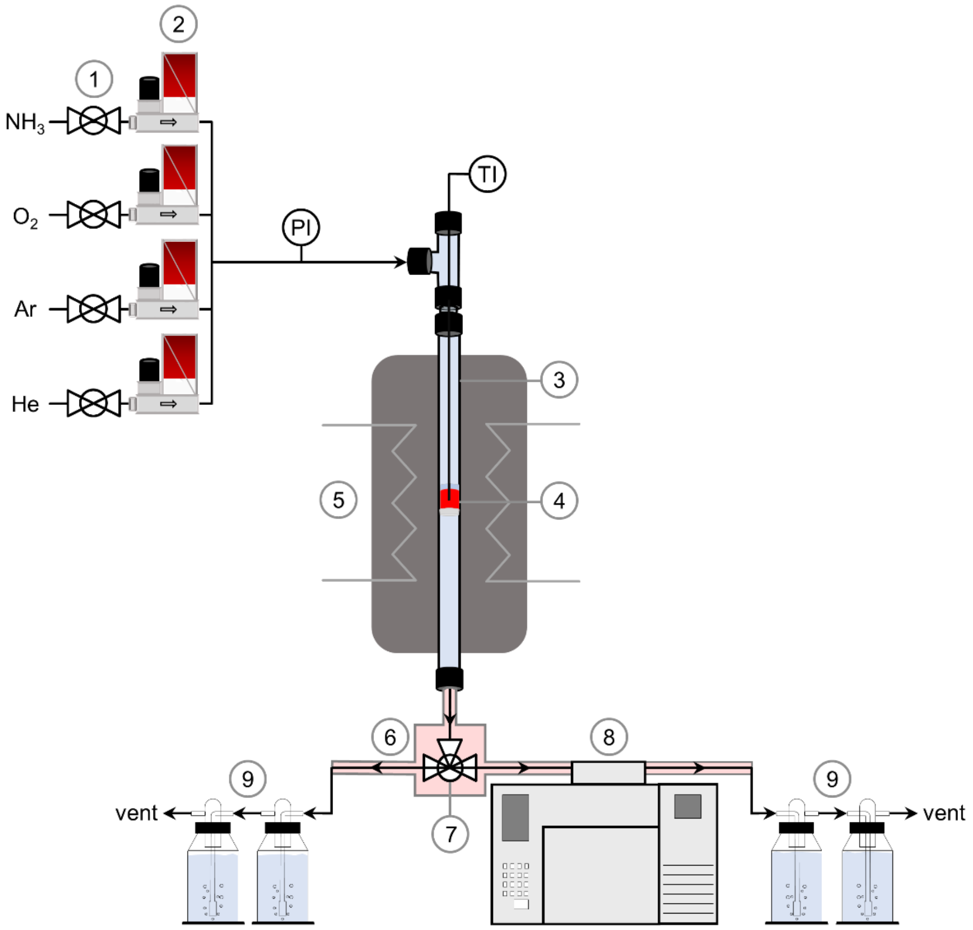

Figure S1. Diagram of the laboratory set-up used for ammonia oxidation. 1: two-way on/off valves, 2: mass flow controllers, 3: quartz reactor, 4: catalyst bed, 5: oven, 6: heat tracing (red background), 7: three-way sampling valve, 8: gas chromatograph coupled to a mass spectrometer (GC-MS), 9: H_2O and H_2SO_4 scrubbers, PI: pressure indicator, and TI: temperature indicator.

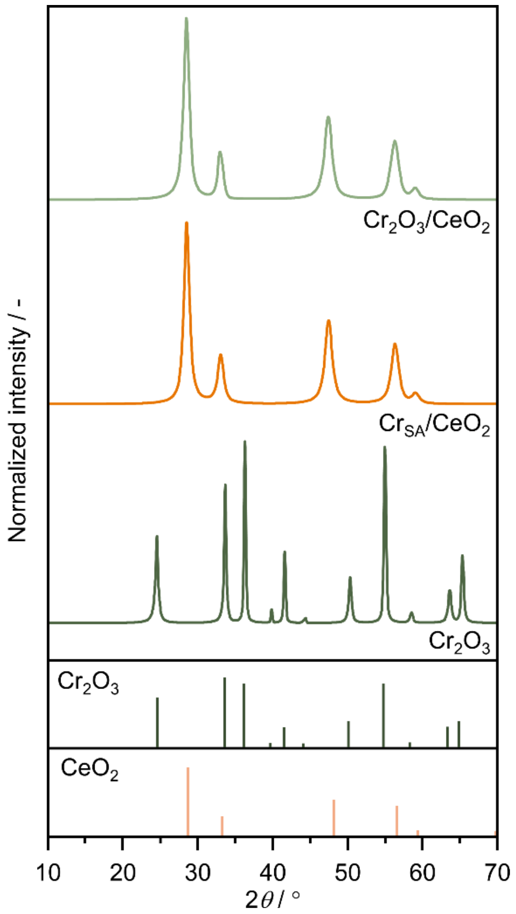

Figure S2. XRD patterns of Cr_2O_3/CeO_2, Cr_{SA}/CeO_2, and Cr_2O_3 particles. Reference patterns of pure phases are shown with vertical lines in the bottom panel. Synthesized Cr_2O_3 particles are crystalline, but their low content on CeO_2 prevents the observation of characteristic reflections.

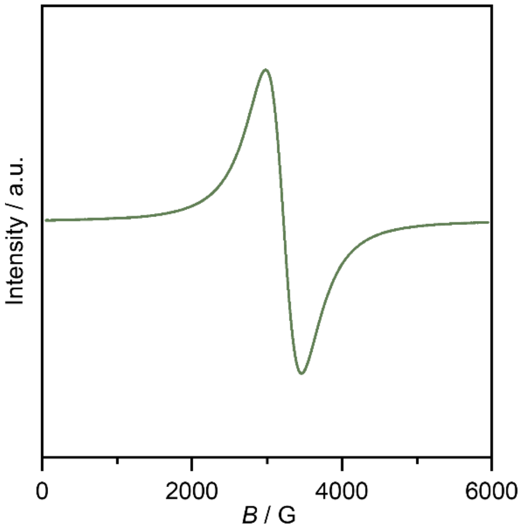

Figure S3. Ex-situ EPR spectrum of Cr_2O_3 particles acquired at room temperature. Cr_2O_3 particles give a broad EPR signal with *g*-factor, $g = 1.985$.

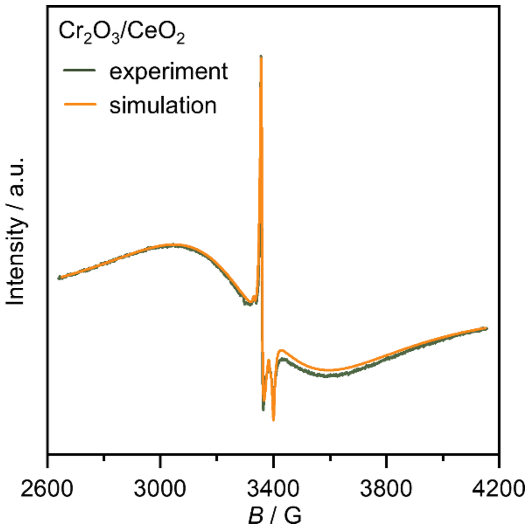

Figure S4. Ex-situ experimental and simulated EPR spectra of Cr_2O_3/CeO_2 at room temperature. Simulation parameters: Cr_2O_3 particles signal: $g = 1.985$, Lorentzian full width at half maximum (FWHM) linewidth: 950 G, Cr_{SA} signal: $g_\perp = 1.9635$, $g_\parallel = 1.9390$; $A_\perp$ (^{53}Cr) = 48 MHz, $A_\parallel$ (^{53}Cr) ≈ 20 MHz, Lorentzian FWHM linewidth = 11 G. EPR spectrum of Cr_2O_3/CeO_2 is dominated by the signal of Cr_2O_3, with contribution from Cr_{SA} comprising only 0.2% of the total signal.

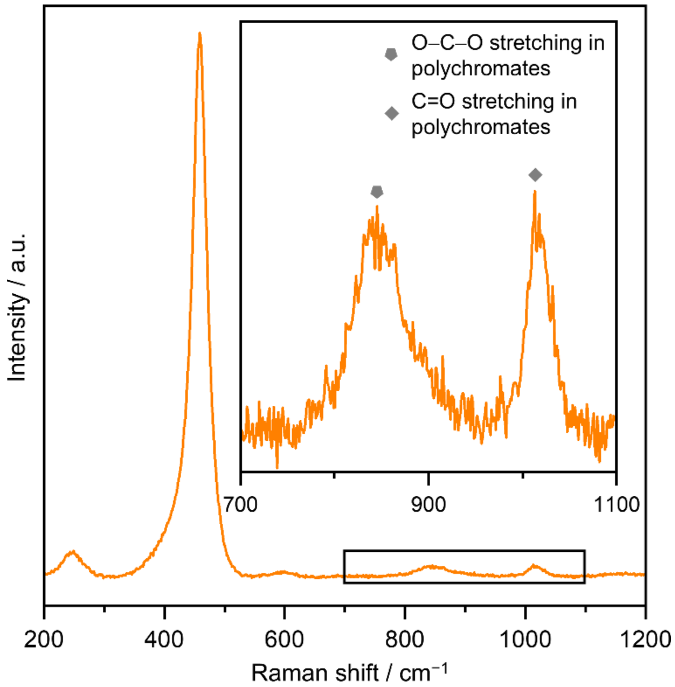

Figure S5. In-situ Raman spectrum of Cr_{SA}/CeO_2 acquired under dehydrated conditions at 473 K.

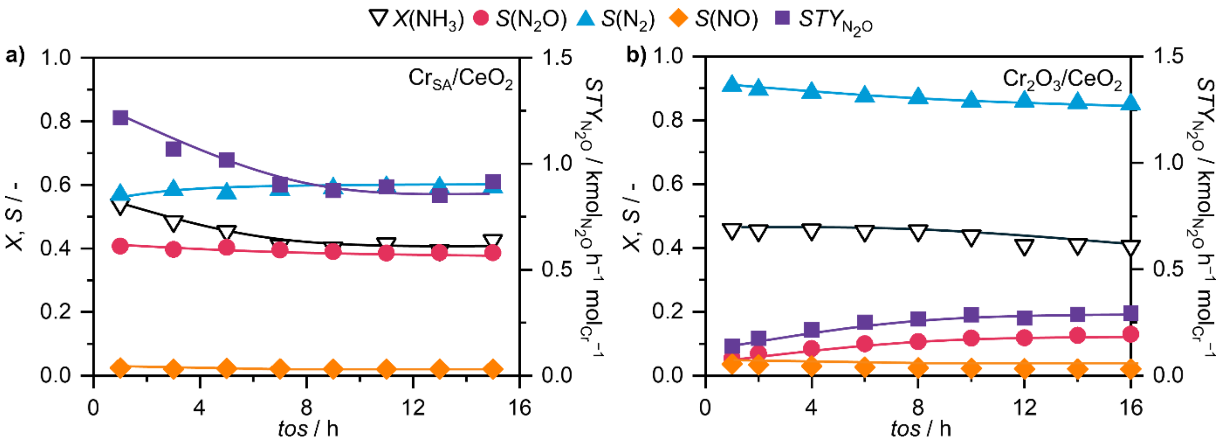

Figure S6. Catalytic performance in NH_3 oxidation, expressed in terms of NH_3 conversion, product selectivity and space-time yield of N_2O of **a)** Cr_{SA}/CeO_2 and **b)** Cr_2O_3/CeO_2 as a function of time-on-stream (tos). Conditions: T_{bed} = 673 K; m_{cat} = 0.01 g; *GHSV* = 600'000 cm^3 h^{-1} g_{cat}^{-1}; *P* = 1 bar; Feed = 8 vol% NH_3, 8 vol% O_2, 4 vol% Ar, 80 vol% He. Different chromium speciation results in drastically different NH_3 oxidation performance over time.

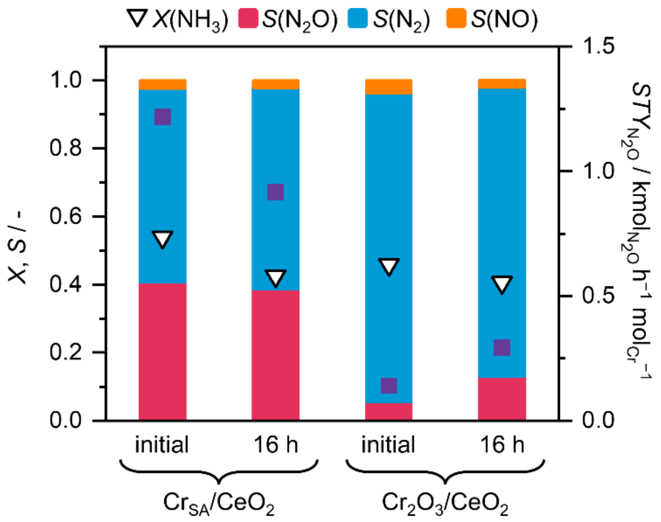

Figure S7. Catalytic performance in NH_3 oxidation, expressed in terms of NH_3 conversion, product selectivity and space-time yield of N_2O of Cr_{SA}/CeO_2 and Cr_2O_3/CeO_2, at the start and end of the stability test depicted in **Figure S6**. Conditions: T_{bed} = 673 K; m_{cat} = 0.01 g; *GHSV* = 600'000 cm^3 h^{-1} g_{cat}^{-1}; P = 1 bar; Feed = 8 vol% NH_3, 8 vol% O_2, 4 vol% Ar, 80 vol% He. The materials show a pronounced difference in initial performance and selectivity changes over time.

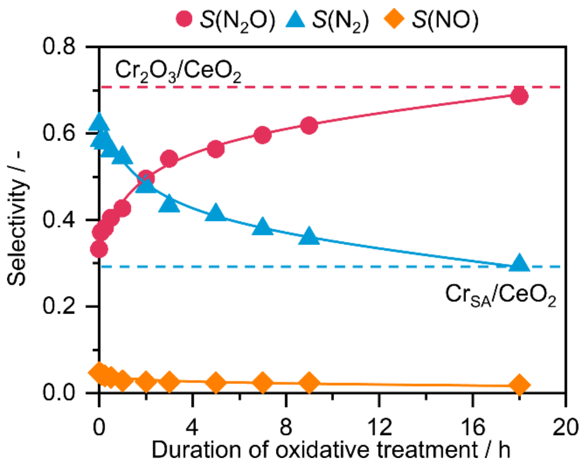

Figure S8. Catalytic performance in NH_3 oxidation, expressed in terms of product selectivity, of Cr_2O_3/CeO_2 after variable duration of an oxidative pre-treatment. Dashed lines correspond to the initial performance of Cr_{SA}/CeO_2 under identical reaction conditions. Conditions: T_{bed} = 673 K; m_{cat} = 0.2 g; $GHSV$ = 15,000 h^{-1} cm^3 g_{cat}^{-1}; P = 1 bar; Feed (reaction) = 8 vol% NH_3, 8 vol% O_2, 4 vol% Ar, 80 vol% He. Oxidative treatment of Cr_2O_3/CeO_2 results in a significant increase in N_2O selectivity, primarily at the expense of N_2.

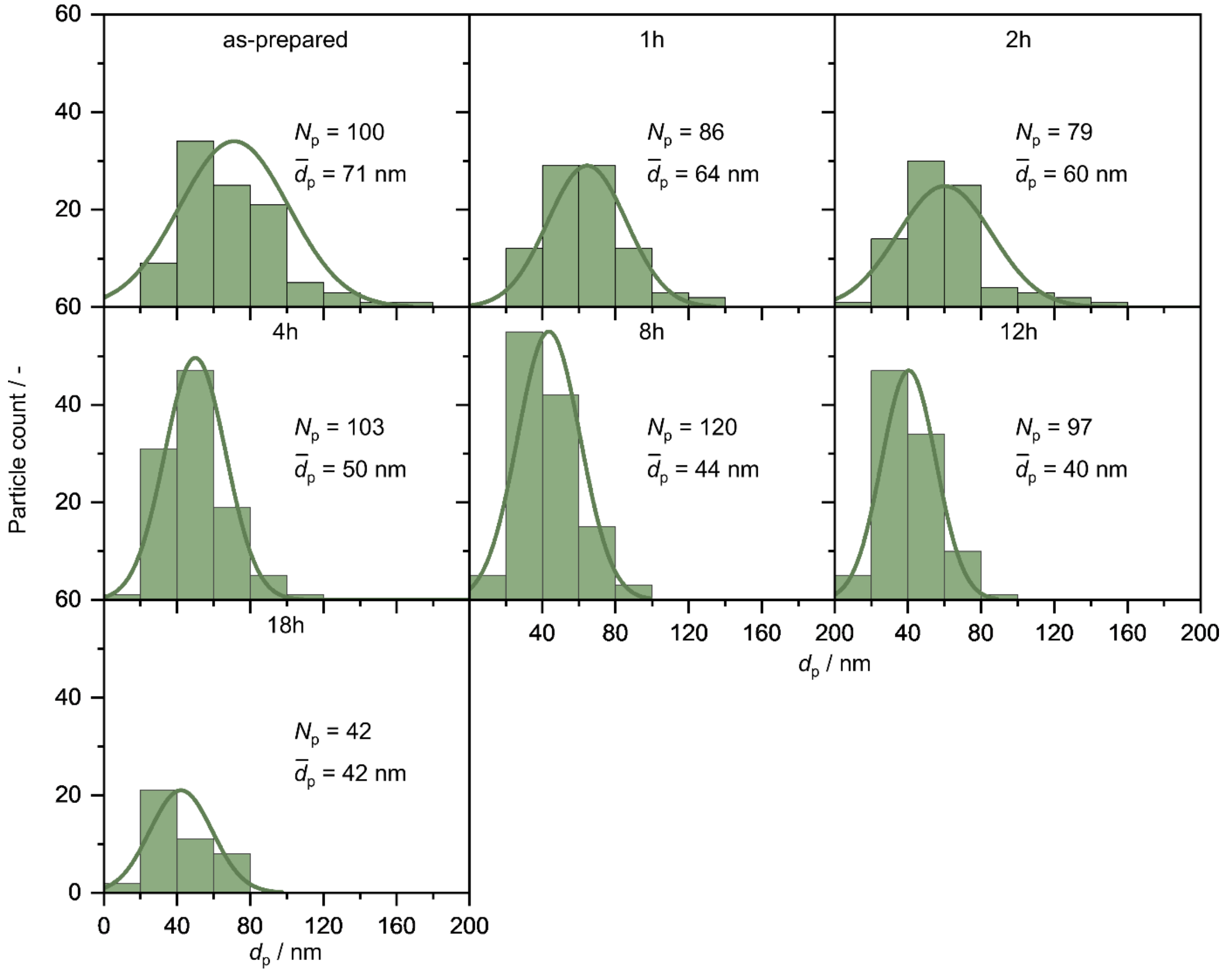

Figure S9. Particle size distribution (PSD) of Cr_2O_3 in Cr_2O_3/CeO_2 after variable duration of an oxidative treatment. N_p corresponds to the total number of particles used to derive the PSD, while $\bar{d}_p$ is the average particle diameter. The average size of Cr_2O_3 particles progressively decreases during the oxidative treatment until the detection limit is reached.

a) b)

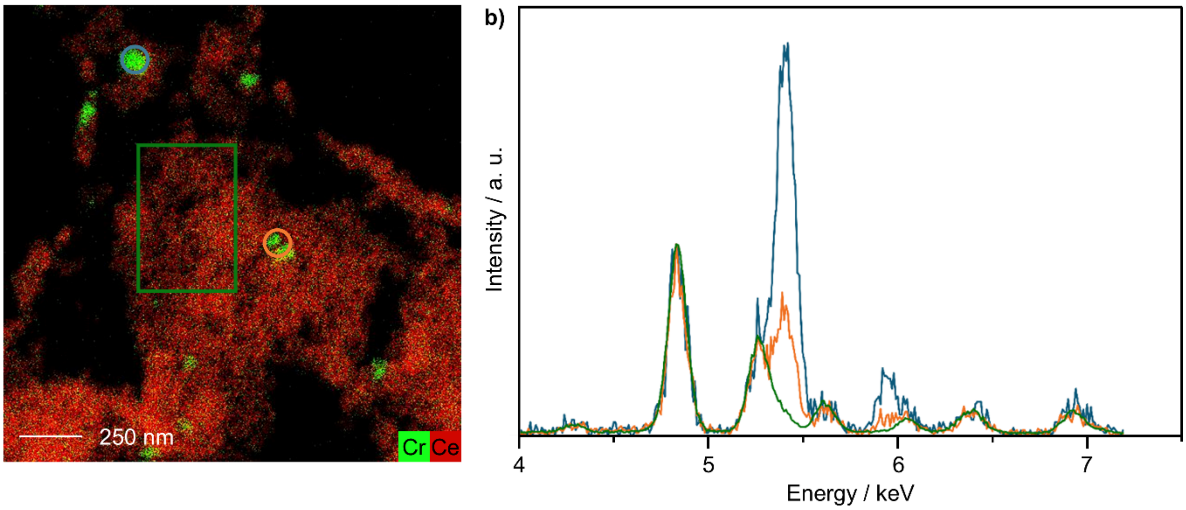

Figure S10. a) Elemental distribution of Ce and Cr obtained by EDXS mapping and **b)** the relevant energy range of EDX spectra extracted at different sites. For comparison, the spectra are normalized to the height of the Ce $L_{\alpha 1}$ peak at 4.84 keV. The Ce $L_{\beta 1}$ (5.262 keV) and Cr $K_{\alpha 1}$ (5.41 keV) lines are close in energy. Therefore, small amounts of Cr are difficult to detect but Cr nanoparticles give a clear signal. For a Cr_2O_3 particle at the edge of a CeO_2 crystal (blue circle), high intensity Cr signal is observed (blue line). For a Cr_2O_3 particle located on the CeO_2 support, a signal typical for Cr is still present (orange circle and line, respectively). The spectrum of an area without visible Cr particles shows the lines of Ce only (green rectangle and line, respectively).

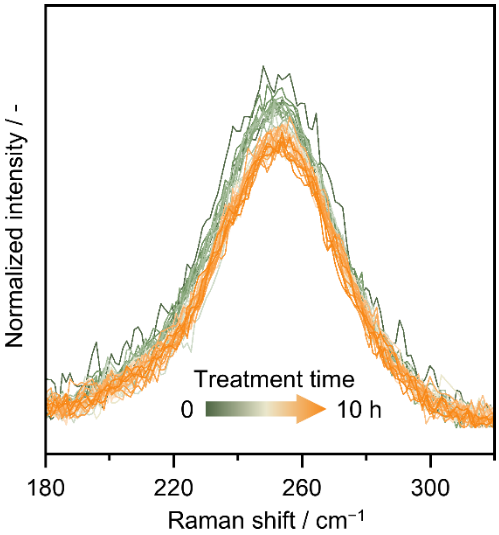

Figure S11. In-situ Raman spectra of Cr_2O_3/CeO_2 during a 10 h oxidative treatment in static air at 673 K, showing the evolution of the band centered at ~250 cm^{-1} due O−Ce longitudinal stretching of atoms in the outermost layers of CeO_2, indicative of partial surface reduction.[5]

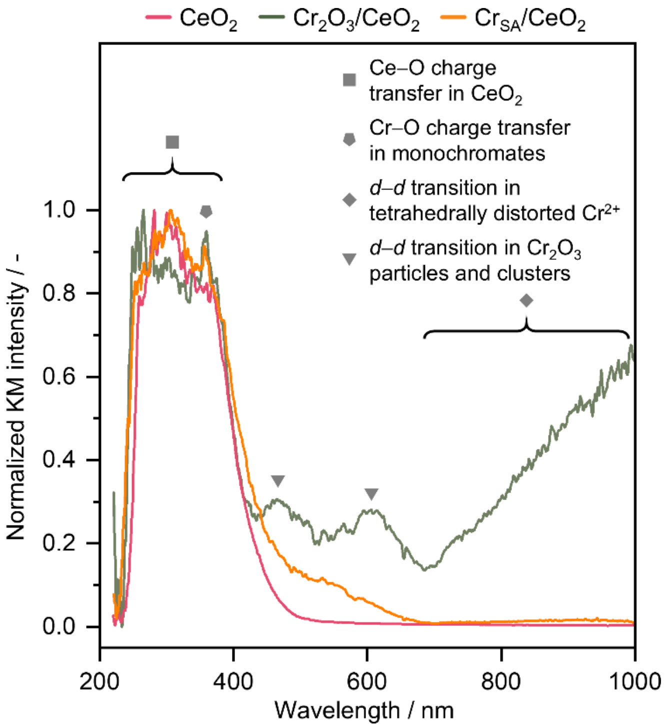

Figure S12. Ex-situ UV-vis DRS spectra of Cr_2O_3/CeO_2, Cr_{SA}/CeO_2 and bare CeO_2, showing distinct spectral features. Strong absorption due to Ce–O charge transfer in the 250-400 nm range obscures chromate-associated features.

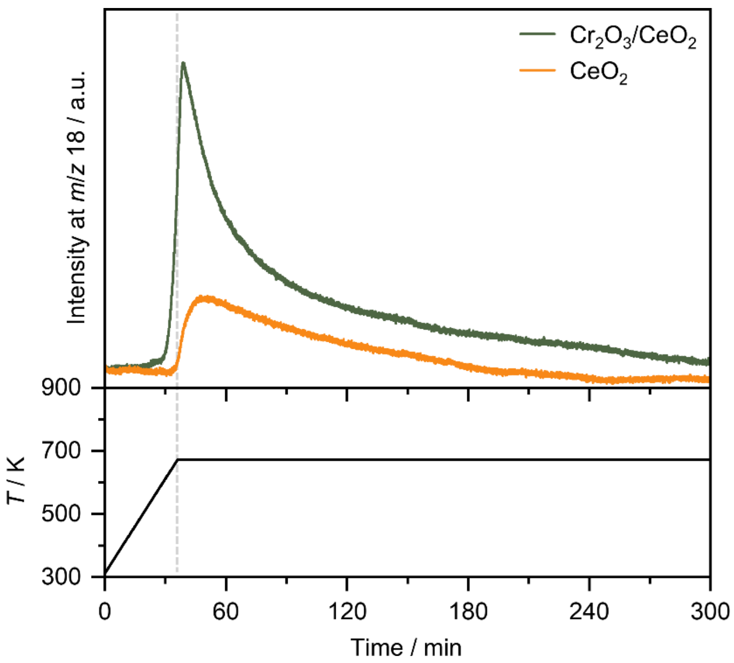

Figure S13. Mass spectrometry profile of the signal at *m*/*z* 18 during a TPO experiment of Cr_2O_3/CeO_2 and the bare CeO_2 support, and the corresponding temperature profile as a function of time. Dehydroxylation of CeO_2 likely occurs during redispersion, leading to the evolution of H_2O.

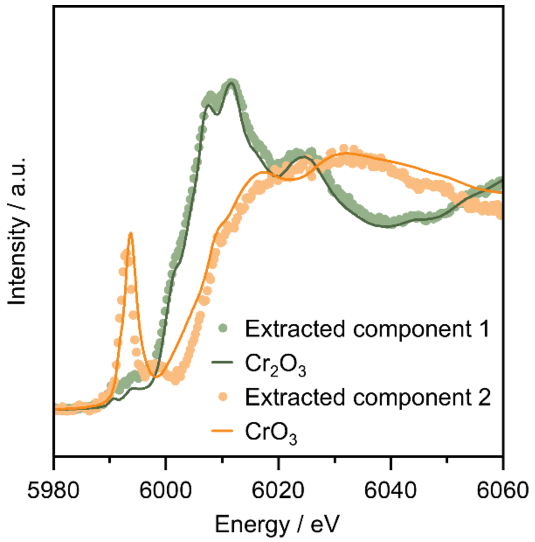

Figure S14. Spectral components depicted in **Figures 6d,** extracted *via* a multivariate curve resolution-alternating least squares (MCR-ALS) algorithm, and the corresponding spectra of chromium standards. The two extracted components show good agreement with Cr_2O_3 and CrO_3 standards, indicating these are two most prevalent chromium species in the sample.

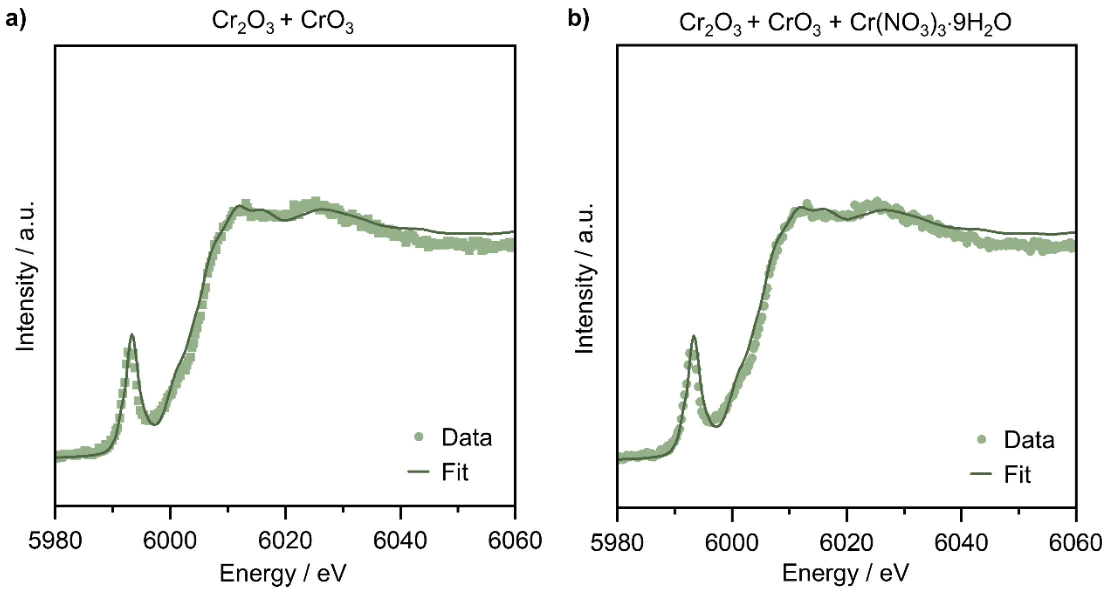

Figure S15. Comparison of the results of MCR-ALS analysis, showing the last averaged data set in **Figure 6c** and the corresponding fit, using **a)** two or **b)** three components and chromium standards as initial guesses. The absence of differences between the two plots suggests that introducing the third component *i.e.*, $Cr(NO_3)_3{\cdot}9H_2O$ does not improve the results.

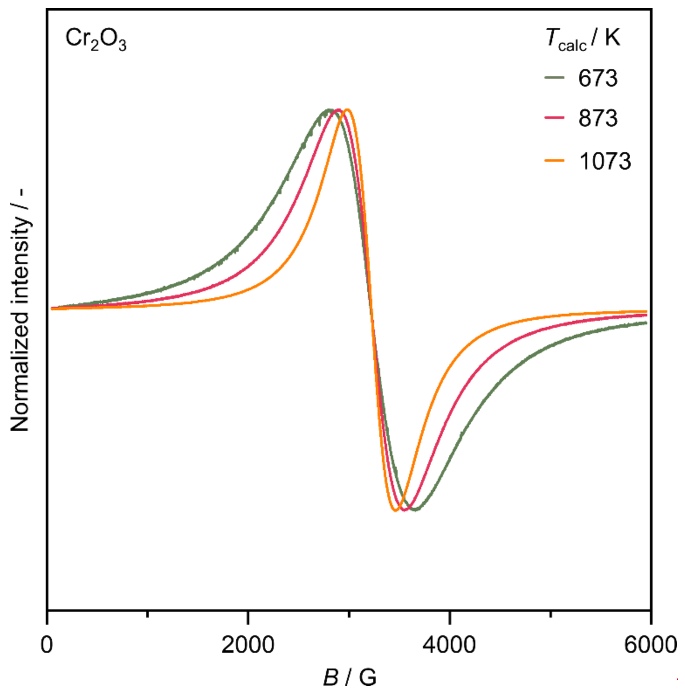

Figure S16. EPR spectra acquired at 673 K of Cr_2O_3 particles calcined at different temperatures, showing the decreasing linewidth with increasing particle size.

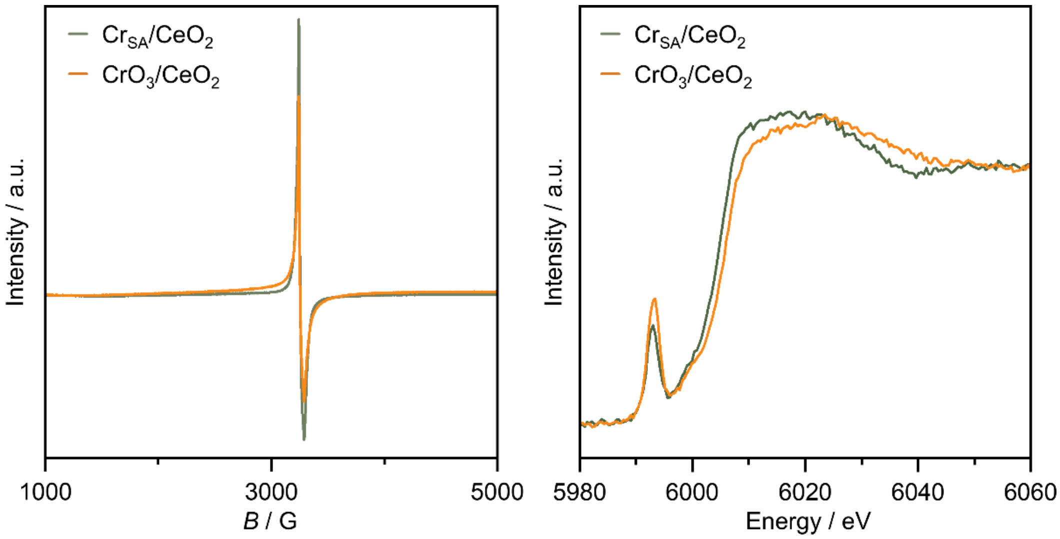

Figure S17. Ex-situ EPR (left) and XANES (right) spectra of Cr_{SA}/CeO_2 and CrO_3/CeO_2 acquired at room temperature. The two samples are highly similar, suggesting that upon calcination the samples derived from different metal precursors converge to a common electronic structure.

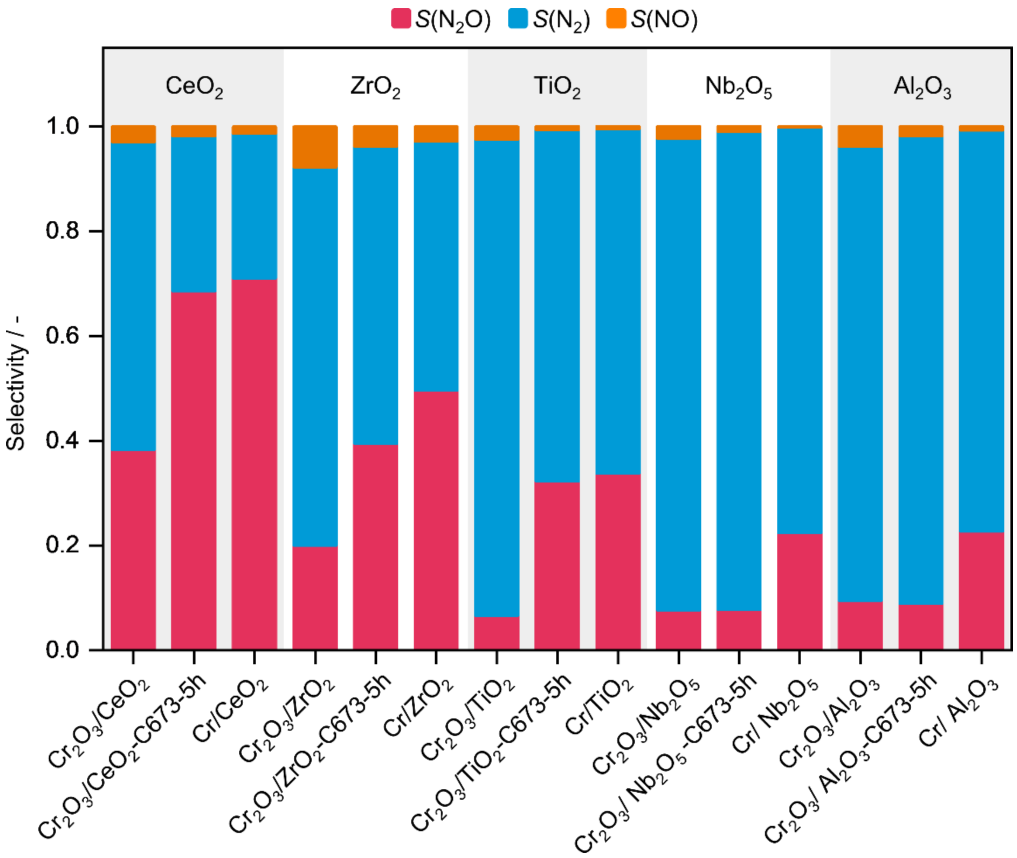

Figure S18. Catalytic performance of chromium catalysts supported on different metal oxides in NH_3 oxidation, expressed in terms of product selectivity at full NH_3 conversion. Conditions: T_{bed} = 673 K; m_{cat} = 0.2 g; $GHSV$ = 15,000 h^{-1} cm^3 g_{cat}^{-1}; P = 1 bar; Feed (reaction) = 8 vol% NH_3, 8 vol% O_2, 4 vol% Ar, 80 vol% He.

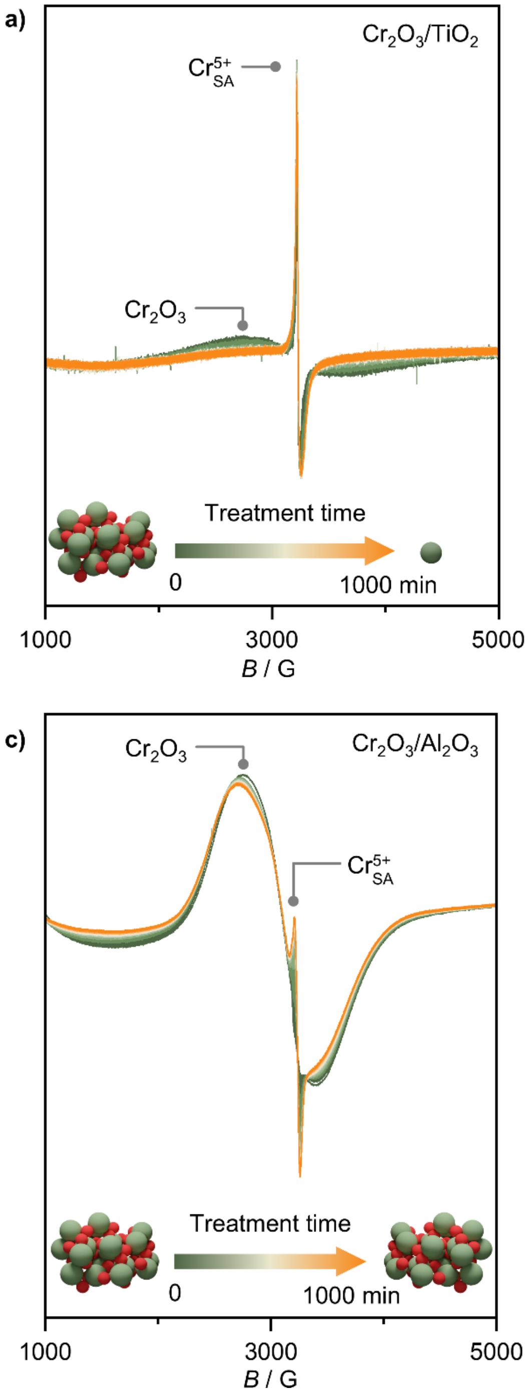

Figure S19. In-situ EPR spectra of **a)** Cr_2O_3/TiO_2, **b)** Cr_2O_3/ZrO_2, **c)** Cr_2O_3/Al_2O_3 and **d)** Cr_2O_3/Nb_2O_5 during an oxidative treatment in static air at 673 K. Spectral changes consistent with chromium redispersion are observed in spectra of Cr_2O_3/TiO_2 and Cr_2O_3/ZrO_2, but little or none are observed in the spectra of Cr_2O_3/Al_2O_3 and Cr_2O_3/Nb_2O_5.

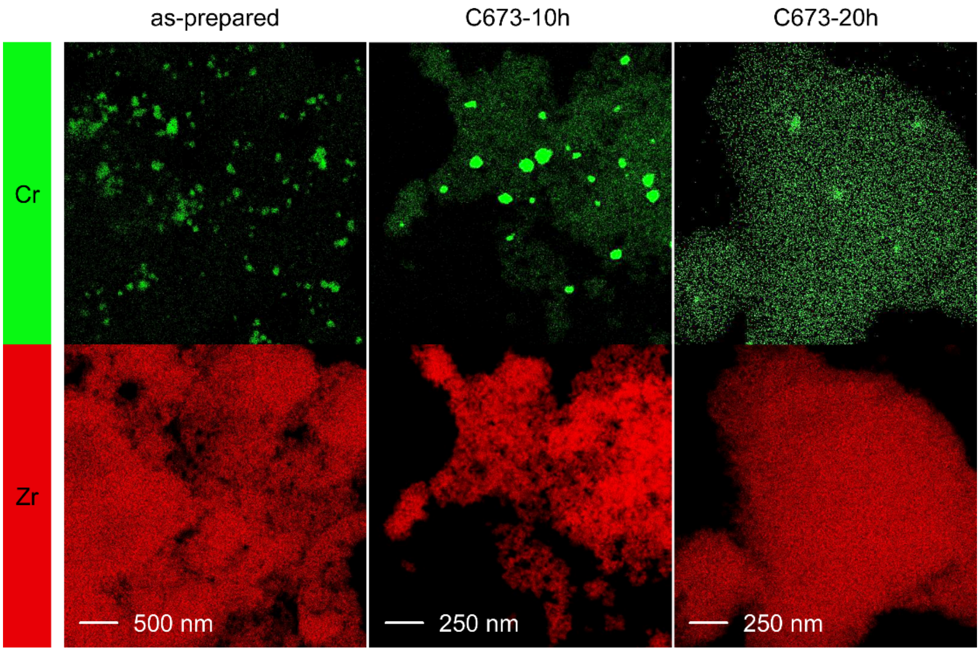

Figure S20. EDX mappings of Cr_2O_3/ZrO_2 in as-prepared form and after variable duration of calcination in static air at 673 K. Progressive increase in chromium dispersion with increasing duration of calcination can be observed.

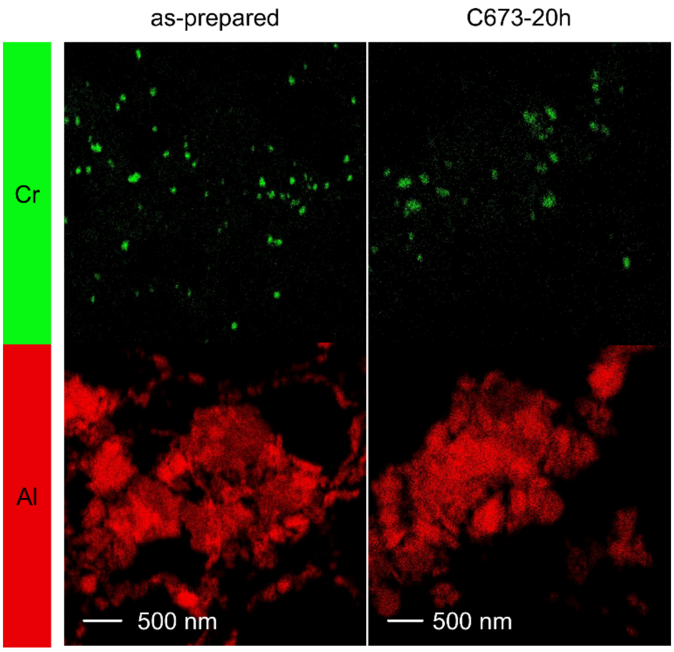

Figure S21. EDX mappings of Cr_2O_3/Al_2O_3 in as-prepared form and after 20 h of calcination in static air at 673 K. Virtually no difference in chromium dispersion can be observed after calcination.

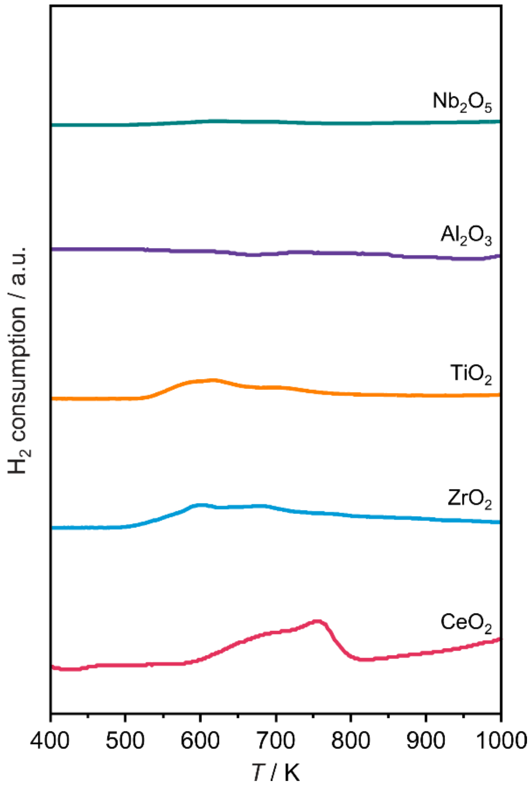

Figure S22. H_2-TPR profiles of bare metal oxide supports, showing distinct reducibility.

4. Supplementary references

(1) van Beek, W.; Safonova, O. V.; Wiker, G.; Emerich, H. SNBL, a dedicated beamline for combined in situ X-ray diffraction, X-ray absorption and Raman scattering experiments. *Ph. Transit.* **2011**, *84* (8), 726. DOI: 10.1080/01411594.2010.549944.

(2) Marshall, K. P.; Emerich, H.; McMonagle, C. J.; Fuller, C. A.; Dyadkin, V.; Chernyshov, D.; van Beek, W. A new high temperature, high heating rate, low axial gradient capillary heater. *J. Synchrotron Radiat.* **2023**, *30* (Pt 1), 267-272. DOI: 10.1107/S1600577522009845.

(3) Ravel, B.; Newville, M. ATHENA, ARTEMIS, HEPHAESTUS: data analysis for X-ray absorption spectroscopy using IFEFFIT. *J. Synchrotron Radiat.* **2005**, *12* (Pt 4), 537-541. DOI: 10.1107/S0909049505012719.

(4) de Juan, A.; Jaumot, J.; Tauler, R. Multivariate Curve Resolution (MCR). Solving the mixture analysis problem. *Anal. Methods* **2014**, *6* (14), 4964-4976. DOI: 10.1039/c4ay00571f.

(5) Schilling, C.; Hofmann, A.; Hess, C.; Ganduglia-Pirovano, M. V. Raman spectra of polycrystalline CeO_2: A density functional theory study. *J. Phys. Chem. C* **2017**, *121* (38), 20834-20849. DOI: 10.1021/acs.jpcc.7b06643.
